# Optimizing the Hydrophobic Environment of Zeolite-Based Catalysts for Promoted Application in Heterogeneous Catalysis

**DOI:** 10.3390/molecules30183670

**Published:** 2025-09-09

**Authors:** Junling Zhan, Chi Zhang, Yu Zhang, Mingjun Jia

**Affiliations:** 1 Department of Physical Chemistry, College of Chemistry, Jilin University, Changchun 130012, China; zhanjl_96@163.com (J.Z.); zchi010214@163.com (C.Z.); 2 School of Chemistry and Pharmaceutical Engineering, Jilin University of Chemical Technology, Changchun 132022, China

**Keywords:** zeolite crystals, hydrophobic environment, catalytic application, heterogeneous catalysis

## Abstract

Zeolites, especially aluminosilicate zeolites, have been widely applied in various important heterogeneous catalytic processes. The catalytic properties of zeolites are highly dependent on their topology structure, composition, porosity and surface chemistry. Recent research progress has demonstrated that increasing hydrophobicity of zeolite-based catalysts may play a positive role in enhancing their catalytic performance for some significant catalytic reactions. In this review, we mainly summarize the prominent advances in achieving catalytic performance enhancement by constructing appropriate hydrophobic zeolite-based catalysts. The main focus is on the regulation strategies and promoting mechanisms for optimizing the hydrophobic environment of zeolite-based catalysts. Through this text, we wish to provide some useful guidance on rationally improving the hydrophobicity of the zeolite-based catalysts for advanced applications in sustainable and green chemical processes, and on exploring the challenges that are faced and future directions in the related research fields.

## 1. Introduction

As a family of microporous crystalline materials, zeolites have been widely used as high-performance heterogeneous catalysts or catalyst supports in the fields of chemical industries and environmental treatments [1,2,3]. For meeting the need of sustainable and green chemical development, great efforts have been devoted to modulating the morphology, porosity, composition and surface chemistry of the zeolite crystals [4,5,6,7]. Among these, the adjustment of the hydrophilicity/hydrophobicity of zeolites has become an attractive research subject, since it may significantly affect their physicochemical property and application in adsorption and catalysis [4,8,9,10,11,12].

For the typical aluminosilicate zeolites like ZSM-5, Beta, USY and SSZ-13, it is well known that the hydrophilicity/hydrophobicity is mainly determined by the composition (e.g., Si/Al ratio) and the surface polar hydroxyl groups (e.g., silanol groups) [11,12,13]. In general, higher Si/Al ratio and lower surface hydroxyl group content could result in lower degrees of hydrophilicity (or higher degrees of hydrophobicity). The inherent hydrophilicity of the zeolite catalysts is usually favorable for the activation of polar molecules, thus leading to important applications such as alcohol dehydration and pollutant removal from water [14,15,16,17,18]. However, in many cases, it was found that the intrinsic hydrophilicity of zeolites, which facilitates the adsorption and accumulation of water within their microporous structures, may have a negative influence on the framework stability, and on the accessibility or integrity of the catalytic sites [8,11,19,20,21,22]. Hence, more recent attention has been drawn on the improvement of hydrophobicity of zeolites for promoted application in various heterogeneous catalytic processes, especially for those water-involved reactions [11,23,24,25].

In general, improving the hydrophobicity of zeolites could be achieved by creating a hydrophobic environment of zeolite crystals, either at external crystallite surfaces or within the internal porous voids [21]. Such a hydrophobic recognition is rooted in the understanding of numerous plants and animals in the natural environment, exemplified by the self-cleaning properties of the lotus leaf [26], the slippery films found on Nepenthes pitcher plants [27,28] and the hydrophobic–hydrophilic patterned backs observed in Stenocara beetles [29]. Inspired by nature, researchers have developed a variety of methodologies to construct a suitable hydrophobic environment for zeolites, aiming to improve molecular transport (diffusion) and stabilize the catalyst structure [30,31,32,33,34,35,36]. These include tuning the framework composition of zeolites [37,38,39], post-modification with silylation agents [34,40], introducing other functional groups (metal ingredients) [36,41] or constructing zeolite-based composites (e.g., core–shell structured materials) [35,42,43,44].

In this review, we summarize the significant advancement in appropriately tailoring the hydrophobic environment of zeolite-based materials and their promoted application in various important heterogeneous catalytic processes. The main concern is the regulation strategies for the improvement of hydrophobicity, and the understanding of their positive role in enhancing the catalytic activity, selectivity and stability for a given reaction. Through this text, we wish to provide some useful guidance on rationally fabricating a hydrophobic environment for the zeolite-based catalysts, to meet the needs of sustainable and green chemical development. In addition, the challenges faced and future directions in the related research fields have also been proposed.

## 2. Improving Hydrophobicity by Tuning Framework Composition and Surface Defect of Zeolites

The framework composition of zeolites refers to the chemical constitution and spatial arrangement of TO_4_ tetrahedral units (T = Si, Al, or other heteroatoms) linked by oxygen atoms in the crystalline structure. The changes in framework composition (e.g., Si/Al ratio) endows zeolites with adjustable properties, including acidity, redox and hydrophilicity/hydrophobicity [1,22,45]. In addition, decreasing the surface defect of zeolites is also an effective way to improve the hydrophobicity of zeolites [46]. In this section, we mainly describe some representative examples of improving the hydrophobicity of aluminosilicate and metallosilicate zeolite catalysts by tuning their framework composition and surface defects.

### 2.1. Aluminosilicate Zeolites

Aluminosilicate zeolites with well-defined Brønsted and Lewis acid sites have been widely utilized as efficient heterogeneous catalysts for various acid-catalyzed reactions [1]. However, their inherently hydrophilic character, which mainly originates from the crystal defects (e.g., external and internal silanol groups) and the acidic hydroxyl groups, may also negatively impact the catalytic performance (including hydrothermal stability) in some water-involved catalytic reactions [10,11]. In general, increasing the Si/Al ratio in the zeolitic framework could decrease the hydrophilicity; meanwhile, it increases the hydrophobicity of the aluminosilicate zeolites, and thus may effectively improve the hydrothermal stability of zeolites [11]. However, higher Si/Al ratios could also simultaneously decrease the number of acidic sites (active sites), potentially affecting the catalytic activity of the zeolite catalysts [8,47]. Hence, considerable efforts have been made to balance the framework composition (e.g., Si/Al ratio) and the surface defects in zeolite crystals (e.g., optimizing synthesis, calcination parameters), to meet the needs of some important catalytic reactions, such as the etherification, ketalization and oxidation of volatile organic compounds.

In an early work reported by Corma and coworkers, two series of H-form Beta zeolites with different Si/Al ratios were comparatively studied for the acetalization of the glucose to form alkyl glucoside surfactants. They found that the series derived from dealumination showed enhanced catalytic activity, due mainly to the improved hydrophobicity, which is caused by the decrease in Si–O–Si connectivity defects. The optimized H-form sample with intermediate acidic sites and relatively high hydrophobicity also exhibited better resistance to deactivation than those samples with similar acidity but lower hydrophobicity [48]. Similarly, Shimizu and coworkers reported that H-Beta zeolite with a Si/Al ratio of 75 (H-Beta-75) showed higher catalytic activity and reusability for the hydration of hydrophobic epoxides and alkynes than the samples with other Si/Al ratios [47]. They proposed that the hydrophobicity was a key factor in influencing the catalytic performance of H-Beta zeolites in hydration reactions. The superior performance of H-Beta-75 was mainly related to the stronger hydrophobic interactions between the pores and substrates [47].

In another work reported by Martins and coworkers, they investigated the catalytic properties of ZSM-5 zeolites with different Si/Al ratios (15–140, denoted as MFI-*n*) for the two-phase ketalization reaction of glycerol with acetone [38]. Although the zeolites with lower Si/Al ratios offered higher availability of acid sites, the higher hydrophilicity made them more prone to water adsorption, which could weaken active sites more intensely. On other hand, the sample of MFI-140 with relatively high hydrophobicity exhibited the highest catalytic efficiency, and its catalytic activity could be further improved by ion-exchanging the external acid sites with *n*-hexylammonium cations, due to the further increase in hydrophobicity. These results demonstrated that the intensified hydrophobic character of ZSM-5 zeolites facilitates the approach of acetone at the proximity of active sites, and thus may overcome mass transfer limitations in these heterogeneous acid-catalyzed reactions [38].

**Figure 1:**
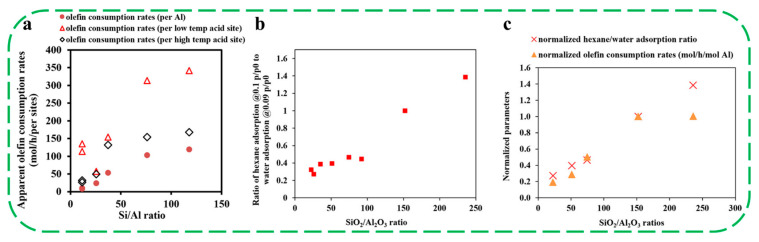
(**a**) Apparent olefin consumption rates per Al, low temperature acid sites, and high temperature acid sites versus Si/Al after 0.25 h reaction time. Reaction conditions: 6.2 g of 1-dodecene, 6.7 g of EG, 0.75 g of catalyst, 125 °C. (**b**) Ratio of hexane adsorption at *p*/*p*_0_ = 0.1 to water adsorption at *p*/*p*_0_ = 0.09 for H-Beta zeolites with different Si/Al ratios. (**c**) Normalized ratio of hexane adsorption to water adsorption and normalized olefin consumption rates as a function of Si/Al. The normalization was based on the data points at Si/Al = 150. Etherification reaction conditions: 6.2 g of 1-dodecene, 6.7 g of ethylene glycol, 0.75 g of catalyst, 125 °C, 0.25 h. Reproduced with the permission of ref. [49]. Copyright 2024, American Chemical Society.

In order to understand the nature of catalytically active sites required for olefin etherification, Lee et al. investigated the effect of acidity and hydrophobicity of H-Beta zeolites (with different Si/Al ratios) on the catalytic performance by combining experimental and computational work [49]. Comparative experiments revealed that there is no correlation between olefin consumption rates, which were normalized either per type of acid site or per total acid sites, and the Si/Al ratio. This finding suggests that the long-chain olefin etherification activity was not driven by the total number of acid sites available in the catalysts (Figure 1a). The hexane and water adsorption isotherms showed that the hydrophobicity of H-Beta zeolites increased gradually with the increase in the Si/Al ratio (Figure 1b). Notably, the trend in olefin consumption rates normalized per Al atom matched well with the trend in the normalized hydrophobicity index, as the Si/Al ratio increased up to 75, indicating a strong correlation between etherification activity per acid site and the hydrophobicity of the zeolite (Figure 1c). The significantly lower etherification activity observed for H-Beta with Si/Al = 12.5 could be attributed to its higher hydrophilicity. This hydrophilic nature likely leads to stronger interactions with ethylene glycol, thereby reducing the local concentration of 1-dodecene on or near the zeolite surface, and ultimately lowering olefin conversion rates. These results reveal clearly that the Brønsted acid sites located inside the micropores of H-Beta with hydrophobic features are the active sites for long-chain olefin etherification with an alcohol [49].

Some recent works also demonstrated that various hydrophobic zeolites may act as effective supports for generating active and stable heterogeneous catalysts [39,50,51,52]. For instance, by using a one-pot synthesis strategy, Tian and coauthors synthesized a series of ZSM-5-encapsulated Pt nanoparticles for the complete oxidation of volatile organic compounds. Compared to the sample prepared by the impregnation method, the encapsulated catalysts exhibited stronger Pt-zeolite interactions and higher Pt dispersion. Among them, Pt/ZSM-5 with Si/Al = 200 showed the best performance, achieving a 98% toluene conversion at 214 °C, along with outstanding stability over 7200 min at 50% relative humidity, even in the presence of 100 ppm 1,2-dichloroethane. The authors attributed the excellent performance to the higher Pt dispersion and the enhanced hydrophobicity at higher Si/Al ratios. In this case, the enhanced hydrophobicity of catalysts facilitated the adsorption of toluene and inhibited the adsorption of halogen compounds (1,2-dichloroethane) on the catalyst; meanwhile, this provided effective protection to the metal active sites against poisoning [39].

In 2024, Peng and coworkers reported that a bifunctional ZnZrO_x_/SSZ-13 catalyst (obtained by physically grinding the ZnZrO_x_ and hierarchical SSZ-13) exhibited exceptional activity in the hydrogenation of CO_2_ to high-value chemicals [50]. Under conditions of sufficient acid sites (Si/Al = 10–25), increasing the hydrophobicity of SSZ-13 zeolites facilitated H_2_O desorption and diffusion into the reaction atmosphere, which in turn increased the CO_2_ conversion rate and decreased selectivity for the byproduct, CO. In contrast, H_2_O cannot diffuse in time on the low-hydrophobicity zeolite, which reduces the rate of methanol to propane, thereby hindering the progress of the reaction and resulting in poor reaction performance. Notably, despite greater hydrophobicity, the SSZ-13 zeolite with a Si/Al ratio of 30 exhibited a decreased CO_2_ conversion and improved CO selectivity due to insufficient acid sites. The authors concluded that, under sufficient acidity, increasing zeolite hydrophobicity could enhance CO_2_ conversion and decrease CO selectivity. Under optimum reaction conditions (390 °C, 3.0 Mpa, a gas hourly space velocity of 1000 mL g^−1^_cat_ h^−1^), the ZnZrO_x_/SSZ-13 (Si/Al = 25) catalyst demonstrated an excellent catalytic performance, achieving a high CO_2_ conversion of 44.7% with a relatively low CO selectivity of 16.7%, leading to 29.7% yield of liquefied petroleum gas and 26.1% yield of propane, respectively.

**Figure 2:**
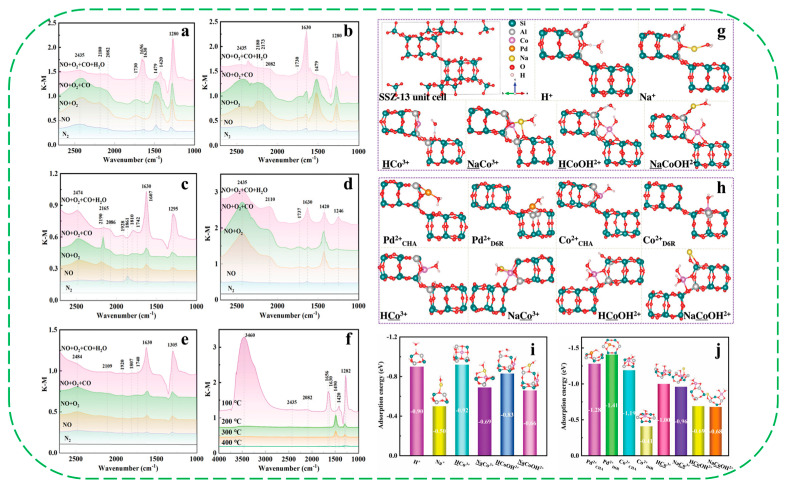
DRIFT spectra after different simulated gas (in the order of N_2_, NO, NO + O_2_, NO + O_2_ + CO, and NO + O_2_ + CO + H_2_O) adsorbed on: (**a**) Co/NaSSZ-13, (**b**) Co-SSZ-13, (**c**) Pd-SSZ-13, (**d**) Na-SSZ-13 and (**e**) H-SSZ-13 for 40 min at 100 °C. (**f**) DRIFT spectra of the temperature programmed desorption over Co/Na-SSZ-13 from 100 °C to 400 °C after saturated with NO + O_2_ + CO + H_2_O for 40 min). The periodic SSZ-13 unit cell contains 36 T-sites and H_2_O molecule adsorption configuration (**g**,**h**) and adsorption energy (**i**,**j**) on different sites. Olive, gray, dark pink, orange, yellow, red and light pink colors represent Si, Al, Co, Pd, Na, O and H atoms, respectively. Reproduced with the permission of ref. [51]. Copyright 2024, John Wiley and Sons.

Chen et al. synthesized Co/Na-SSZ-13 zeolite via a one-step method using N,N,N-Trinethyl-1-ammonium adamantane and Co-tetraethylenepentamine as the template and Co precursor, without the need of an ammonia exchange process [51]. The Co/Na-SSZ-13 zeolite exhibited an outstanding NO storage capacity of 212 μmol g_cat_^−1^ under humid conditions (3% H_2_O *v*/*v*), significantly surpassing that of Co-SSZ-13 (82 μmol g_cat_^−1^) and Pd-SSZ-13 (94 μmol g_cat_^−1^) prepared by the conventional two-step route. Through in suit diffuse reflectance infrared Fourier transform spectroscopy (DRIFTS, Figure 2a–f) and density functional theory (DFT) calculations (Figure 2g–j), the authors attributed the enhanced NO storage capacity under humid conditions to two primary aspects: (1) Na^+^ ions decrease the amount of Brønsted acid sites (Si–OH–Al), thereby reducing the affinity of the Co/Na-SSZ-13 with H_2_O; and (2) Na^+^ ions shield the negative charge of the Al center and weaken the local electric field strength of the Al and Co centers, thus weakening the adsorption energy of the H_2_O molecule and improving the hydrophobicity of zeolite.

**Figure 3:**
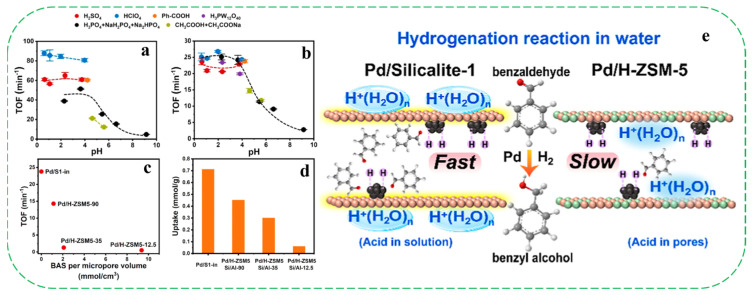
Turnover frequency (TOF) of benzaldehyde hydrogenation catalyzed by (**a**) Pd/SiO_2_ and (**b**) Pd/S1-in at different pH values. (**c**) TOF of Pd/H-ZSM5 with different BAS concentrations in the hydrogenation reaction of benzaldehyde. (**d**) Benzaldehyde uptake on different zeolites. (**e**) the comparison of benzaldehyde hydrogenation mechanism over Pd/Silicalite-1 and Pd/H-ZSM-5 in aqueous phase. Reproduced with the permission of ref. [52]. Copyright 2025, American Chemical Society.

Very recently, Shi et al. reported the synthesis of two Pd/S-1-in and Pd/H-ZSM-5 catalysts, in which Pd nanoparticles were encapsulated within the pores of silicalite-1 and H-ZSM-5 (Si/Al = 12.5–90), respectively [52]. As shown in Figure 3a,b, the hydrogenation of benzaldehyde over hydrophobic Pd/S1-in exhibited a pronounced dependence on the aqueous pH, showing the turnover frequency (TOF) increased by one order of magnitude as the pH decreased from nine to four. In contrast, Pd/H-ZSM-5 catalysts (Si/Al = 12.5–90) consistently showed lower TOFs than Pd/S-1-in (TOF values of about 24 min^−1^), with activity decreasing progressively as the concentration of Brønsted acid sites (BAS) increased. For instance, Pd/H-ZSM-5-12.5, possessing the highest BAS concentration, exhibited the lowest TOF (about 0.8 min^−1^, Figure 3c,d). The authors attributed the enhanced hydrogenation activity of Pd/S-1-in in acidic environments to the reduced electron density of Pd particles, which can weaken hydrogen binding and promote the activation of adsorbed hydrogen. As for Pd/H-ZSM-5, however, the H_3_O^+^ ions located directly within the micropores near Pd sites compete with benzaldehyde for pore occupancy, thus largely decreasing the uptake of benzaldehyde in the pore and reducing the TOF. This competitive effect becomes more pronounced with increasing BAS concentrations. Additionally, they proposed that the silanol groups (Si–OH) that exist on hydrophobic silicalite-1 can dissociate in water to form an acid/conjugate base pair (Si–OH/Si–O^−^), forming an acidic microenvironment within the pores that reflects the bulk aqueous pH. This local acidity is believed to enhance the hydrogenation activity of the encapsulated Pd particles (Figure 3e).

The above results clearly demonstrate that adjusting the Si/Al ratio is an effective way to modulate the hydrophobic environment of zeolites and thereby may significantly improve their catalytic performance in some important catalytic reactions. As a matter of fact, variations in the Si/Al ratio could also affect other physicochemical properties of the zeolites, such as the acidity, porosity, particle size and surface defects. These complexities make it difficult to determine whether the hydrophobicity or acidity (even other factors) plays the dominant role in influencing the catalytic performance of the zeolite-based catalysts, thereby hindering the understanding of the actual reaction mechanisms (or structure-activity relationships). Therefore, it will be necessary in future to design more systematic research methodologies for studying the impact of hydrophobicity on the catalytic properties in a special catalytic process, for instance, to integrate the well-designed comparative experiments, advanced characterization techniques (e.g., in situ characterization and isotope labeling) and theoretical computational methods for the purpose of accurately identifying the intrinsic properties of active sites, and thereby gain deeper insights into the mechanistic role of zeolite hydrophobicity in catalytic reactions. In addition, synthetic optimization strategies (e.g., morphology control, crystal facet engineering and the introduction of mesoporous structures) may offer further opportunities to enhance the hydrophobicity, alleviate diffusion limitations, and ultimately gain deeper insights into the intrinsic structure–performance relationships of the zeolite-based catalysts.

### 2.2. Metallosilicate Zeolites

Metallosilicate zeolites are a class of microporous materials formed via the isomorphous substitution tetravalent or high-valent metal ions (e.g.,Ti^4+^, Sn^4+^, Zr^4+^, W^6+^, Hf^4+^, Ge^4+^) into pure silica frameworks. These materials have drawn considerable attention since the appearance of titanium silicalite-1 (TS-1, with MFI topology) in 1983 [20]. Metallosilicate zeolites typically possess a water-tolerant ability and are capable of being served as promising heterogeneous catalysts involving aqueous reactions [20,22]. In this section, we summarize the recent progress on the synthesis and catalytic application of various hydrophobic metallosilicate zeolites (e.g., titanium-containing zeolite, tin-containing zeolites and tungsten-containing zeolites).

Metallosilicate zeolites are commonly obtained by a traditional hydrothermal method in the presence of various organic structure-directing agents (OSDA). Recently, more efforts have been devoted to the synthesis of various metallosilicate zeolites with different structures, porosity and morphology through more simple and efficient ways [53,54,55,56,57,58,59,60,61,62,63,64,65,66,67,68,69,70]. For instance, in 2022, Kubota and coworkers reported for the first time that an OSDA-free Al-Beta zeolite could be converted to a Ti-Beta catalyst via deep dealumination with acid treatments and followed by Ti-incorporation [53]. Characterization by water adsorption isotherms and ^29^Si NMR revealed that the resultant (Ti)-Beta was more hydrophobic than those samples synthesized in presence of OSDA. Catalytic tests showed that (Ti)-Beta exhibited the highest turnover number (TON = 105) and product yield (11.8%) in the oxidation of phenol with H_2_O_2_. The excellent catalytic performance of the (Ti)-Beta should be mainly ascribed to an appropriate balance between hydrophilicity and hydrophobicity realized by increasing hydrophobicity of the zeolite catalysts, which can effectively avoid the formation of undesirable oligomers, thereby increasing the product yield.

**Figure 4:**
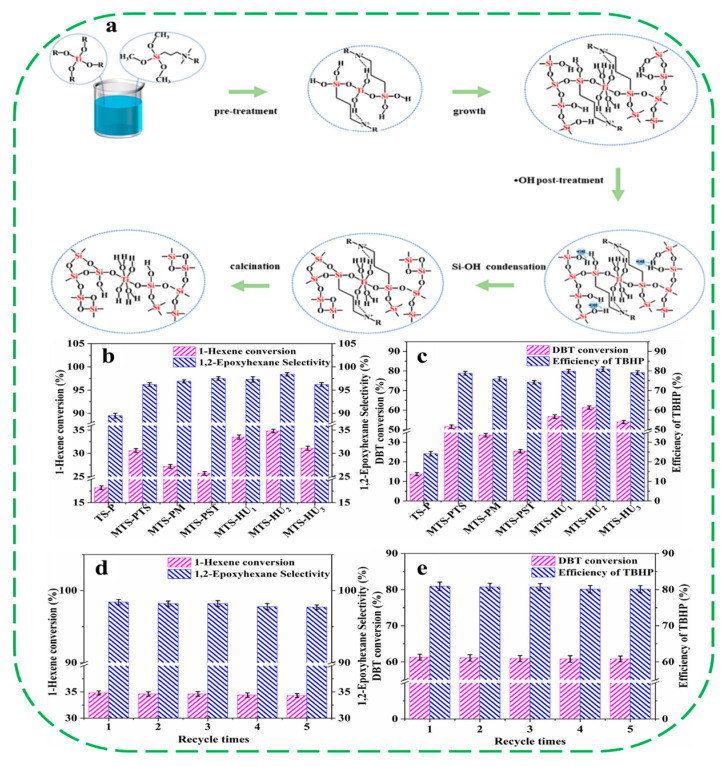
(**a**) Proposed evolution of TiO_6_ in TS-HU_2_. (**b**–**e**) 1-Hexene conversion and 1,2-epoxyhexan selectivity at 2 h (**b**), as well as DBT conversion and TBHP efficiency at 5 min (**c**) over different catalysts, and the recycle test of MTS-HU_2_ in 1-hexene epoxidation at 2 h (**d**) and oxidative desulfurization of DBT at 5 min (**e**). Reproduced with the permission of ref. [54]. Copyright 2023, Elsevier.

In 2023, Diao and coworkers reported that the hierarchical TS-1 zeolites (denoted as MTS-HUs) with high hydrophobicity and a high concentration of accessible TiO_6_ could be obtained by sequential organosilane (TPOAC)-assisted pretreatment and dry-gel conversion in presence of meso-porogens (CTAB) [54]. During the pre-treatment, TPOAC interacted with Ti species, and the resulting TPOAC-Ti component not only acted as the co-mesoporogen in conjunction with the surfactant, but also as a stabilizing agent for the Ti species during the dry-gel conversion process. This approach effectively limited TiO_2_ formation and induced preferential distribution of TiO_6_ units near the mesopore surfaces, thereby significantly increasing TiO_6_ accessibility. Subsequently, hydroxyl radical post-treatment accelerated the condensation of Si–OH groups on the mesopore surface and enhanced the hydrophobicity of the meso-TS-1, while exerting a small influence on Ti coordination (Figure 4a). Compared to conventional TS-P, the optimized hierarchical MTS-HU_2_ catalyst displayed outstanding catalytic performance in both 1-hexene epoxidation and oxidative desulfurization, achieving considerable conversion improvements (Figure 4b,c). After five cycles, the catalytic performances of MTS-HU_2_ were all maintained, indicating its excellent catalytic stability (Figure 4d,e). The superior catalytic activity of MTS-HU_2_ could be mainly attributed to the synergistic effect of its hierarchical porosity, high accessibility of TiO_6_ sites, and enhanced hydrophobicity.

More recently, Sun’s group systematically synthesized a series of Au-loaded hierarchical TS-1 zeolites (Au/MTS-1) via a two-step recrystallization approach, combined with the introduction of small amounts of surfactants with different carbon chain lengths (C6–C16) [55]. As shown in Table 1, Au/MTS-1-C16 exhibited the highest epoxide (PO) selectivity (~90%) and stability (25 h) for propylene epoxidation with H_2_ and O_2_, outperforming the catalysts of Au/MTS-1-C8 (~60%, 6 h), Au/MTS-1-C10 (~60%, 13 h) and Au/MTS-1-C12 (~78%, 17 h), which were derived from the surfactants with shorter carbon chains. The improvement of the catalytic performances could be mainly attributed to the enhanced diffusion ability and the increased hydrophobicity of the MTS-1-C16 support, as proved by the increased hierarchical factor (HF) of 0.084 and the decreased terminal silanols (Q^3^/Q^4^ = 8.92%), which can restrain the ring-opening reaction of PO and formation for aromatic carbonaceous deposits.

In a subsequent work, they also developed Au/TS-1 catalysts, utilizing an alkaline-assisted excessive impregnation (AEI) method. This approach facilitated Cl^−^ removal from the Au impregnation precursor, achieved >95% Au nanoparticle loading efficiency and prevented abnormal nanograin growth. Compared with the catalysts prepared by incipient wetness impregnation (IWI) and deposition-precipitation (DP), the Au/TS-1-AEI catalyst exhibited much higher C_3_H_6_ conversion (8.0%), PO selectivity (96.7%) and exceptional stability (>100 h) in gas-phase propylene epoxidation. The superior performance was mainly attributed to the enhanced hydrophobicity, optimized surface acidity and the formation of an oxygen-containing free radical environment [56].

In 2024, Zhu and authors investigated the ketonization mechanism of propionic acid over TS-1 and Ti-Beta zeolites, with particular emphasis on the influence of pore topology and hydrophobicity on the catalytic performance. Characterization results showed that both TS-1 and Ti-Beta (Si/Ti = 40) exhibited a similar acidity; However, TS-1 was more hydrophobic than the silanol-rich Ti-Beta. In the ketonization of propionic acid at 350 °C, TS-1-40 exhibited higher catalytic activity, selectivity, stability and greater resistance to H_2_O compared to Ti-Beta-40. The study revealed that molecularly adsorbed monodentate propionic acid is the most abundant reactive intermediate, and the rate-determining step involves C–C coupling between two co-adsorbed propionic acid molecules at the same tetrahedral Ti center for both zeolites. The superior catalytic performance of TS-1 is attributed to the combined effects of pore topology-induced shape selectivity and the entropic contribution to the free activation energy arising from differences in zeolite hydrophobicity [57].

The first report on isomorphic substitution of Si by Sn was presented by Ramaswamy and coauthors, who conducted the direct synthesis of Sn–MFI zeolite in alkaline media. Using IR spectroscopy and X-ray photoelectron spectra characterizations, they confirmed that Sn atoms were successfully incorporated into the MFI zeolite framework [71]. Later, Wu and coauthors hydrothermally synthesized highly hydrophobic Sn–Beta zeolites with high framework Sn contents (denoted as Sn–Beta–Re) by employing a structure reconstruction strategy [58]. The resultant Sn–Beta–Re-150 exhibited significantly higher catalytic activity (Conv. = 26.9%, TOF = 195 h^−1^, STY = 3.6 h^−1^) in the Baeyer–Villiger (B-V) oxidation of 2-adamantanone with aqueous hydrogen peroxide compared to Sn-Beta-F-150 synthesized via a fluoride-medium route (Conv. = 16.6%, TOF = 123 h^−1^, STY = 2.2 h^−1^). The superior performance was mainly attributed to its distinct features, including smaller crystal size and enhanced hydrophobicity. Furthermore, Sn–Beta–Re-30 (Conv. = 61.6%, TOF = 98 h^−1^, STY = 8.3 h^−1^) with excellent hydrophobicity still exhibited higher performances than the two post-synthesized Sn–Beta zeolites of Sn–Beta–GPS-36 (Conv. = 48.6%, TOF = 84 h^−1^, STY = 6.4 h^−1^) and Sn–Beta–SSIE-30 (Conv. = 47.3%, TOF = 73 h^−1^, STY = 6.2 h^−1^) at similar Sn contents. After the fourth run, the used catalyst was regenerated by calcination in air. The activity for Sn–Beta–Re and Sn–Beta–F was nearly fully restored with retention rates of 96%, whereas Sn–Beta–GPS-36 and Sn–Beta–SSIE-30 showed only partial recovery, with retention values of 78% and 72%, respectively (Table 2). Characterization results indicated that the coordination state of Sn remained almost unchanged in Sn–Beta–Re-30. In contrast, a noticeable increase in extra-framework Sn species was detected in both Sn–Beta–GPS-36 and Sn–Beta–SSIE-30. The above results suggested that Sn–Beta–Re catalyst exhibited outstanding water tolerance and remarkable structural stability under high-temperature calcination, as evidenced by their excellent reusability with preserved Sn coordination states after multiple reaction cycles.

By using polydiallydimethylammonium chloride (PDAD) as a mesoporous structure-directing agent, Tang et al. synthesized hierarchical Sn–Beta–H zeolites, which started from dealumination-derived siliceous Si–Beta and subsequently self-assembled the resulting silica fragments with a Sn precursor in presence of NH_4_F [59]. TG and DRIFT spectra revealed that Sn–Beta–H4 sample possessed higher hydrophobicity than the post-synthesized meso-Sn–Beta samples. The hierarchical Sn–Beta–H4 exhibited much higher catalytic activity in the transformation of glucose into methyl lactate (ML), which could be associated with its hierarchical structure, improved hydrophobicity, and the Lewis acidity. Moreover, Sn–Beta–H4 showed superior resistance to catalytic deactivation and could be fully regenerated without loss of activity through simple calcination in air after reuse.

In addition, Zhang et al. synthesized a novel Sn-containing zeolite with MSE topology (Sn-MCM-68) by treatment of the dealuminated MCM-68 with SnCl_4_ vapor [60]. The Sn species were mainly incorporated into the zeolite framework through reactions between SnCl_4_ and the Si–OH groups in hydroxyl nests. The resultant Sn-MCM-68 exhibited enhanced catalytic performance in the Baeyer–Villiger oxidation of 2-adamantanone and/or cyclohexanone, outperforming Sn-BEA, Sn-MWW, and Sn-MFI catalysts. The excellent catalytic performance of Sn-MCM-68 could be mainly attributed to its three-dimensional 12 × 10 × 10-membered ring pore-opening structure and relatively higher hydrophobicity. Using a similar approach, Zhu and coworkers prepared hierarchical Sn-Y zeolite by a simple solid-state ion-exchange method. Compared with other four Sn-containing zeolites (Sn–MFI, Sn–MWW, Sn–Beta and Sn–MCM-41), the Sn–Y sample showed higher catalytic activity and stability in Baeyer–Villiger oxidation, irrespective of using aqueous hydrogen peroxide or bulky tert-butyl hydroperoxide as the oxidant. By combining a variety of characterization and comparative experiment results, the authors proposed that the enhanced catalytic performance of Sn–Y zeolite was ascribed to its higher hydrophobicity and opened a channel system [61]. In addition, both Sn–MCM-68 and Sn–BEA catalysts showed a gradual decline in TOF over multiple reaction cycles when regenerated solely by chlorobenzene washing. For Sn–MCM-68, the Sn coordination environment remained largely intact after reaction. Its deactivation was mainly due to pore blockage by heavy compounds that chlorobenzene could not remove, which is a reversible process as calcination could nearly fully restore activity. In contrast, Sn–BEA underwent irreversible deactivation even after calcination, resulting from inherent framework defects that promoted hydrolysis by water, leading to Sn leaching and extra-framework Sn formation.

**Figure 5:**
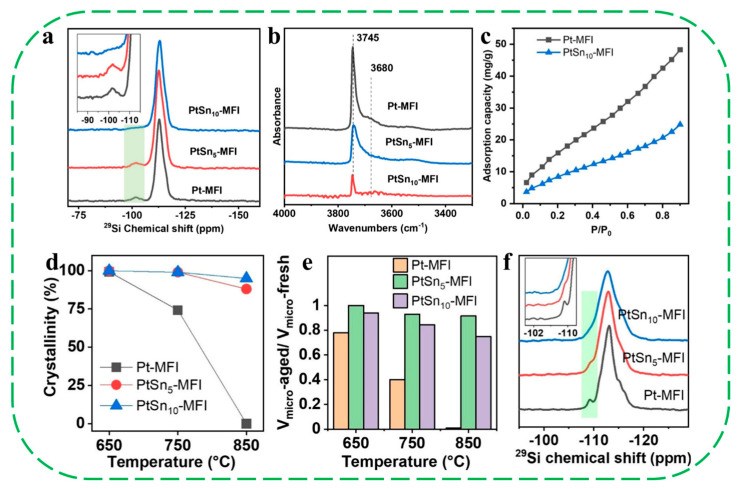
(**a**–**c**) Structural characterization of Pt–MFI and various PtSn–MFI. (**a**) ^29^Si MAS NMR spectra and (**b**) IR spectra of Pt–MFI and various PtSn–MFI with different Sn contents. (**c**) Water isotherm adsorption profiles of Pt–MFI and PtSn_10_–MFI samples at 25 °C. (**d**–**f**) Stabilization of the MFI zeolite framework via the incorporation of Sn into the MFI zeolite. (**d**) Relative crystallinity of Pt–MFI and PtSn–MFI samples obtained after hydrothermal treatments at 650–850 °C. The relative crystallinity was calculated from the XRD patterns of the Pt zeolite samples. (**e**) The ratio of retained micropore volumes of Pt–MFI and PtSn–MFI samples after hydrothermal treatments at 650–850 °C. The micropore volumes were measured by Ar adsorption–desorption isotherms. (**f**) ^29^Si solid-state NMR spectra of Pt–MFI and PtSn–MFI samples after hydrothermal treatments at 650 °C. Reproduced with the permission of ref. [62]. Copyright 2025, American Chemical Society.

**Figure 6:**
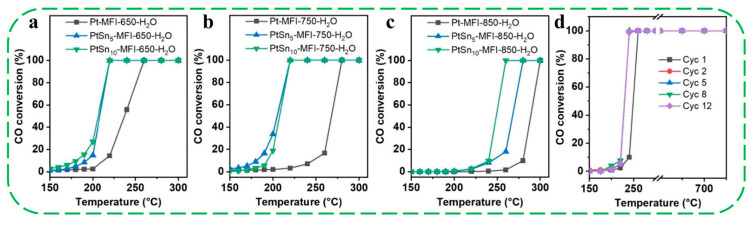
Catalytic performance of the pristine and hydrothermally treatment Pt–zeolite samples for the CO oxidation reaction. (**a**–**c**) CO conversion profiles of Pt–MFI and PtSn–MFI derived from hydrothermal treatments at (**a**) 650 °C, (**b**) 750 °C, and (**c**) 850 °C. (**d**) Stability tests of the PtSn_10_–MFI-850-H_2_O catalyst. The CO oxidation test is performed in the atmosphere, consisting of 1% CO, 16% O_2_, and balanced N_2_, with a gas-hour space velocity of 60,000 mL/g/h. For the consecutive stability tests from room temperature to 750 °C, 4% H_2_O was added to the reaction feed. Reproduced with the permission of ref. [62]. Copyright 2025, American Chemical Society.

More recently, Li et al. developed a strategy to enhance the hydrothermal stability of the Pt-MFI zeolites by incorporating Sn into the zeolite framework (denoted PtSn_x_-MFI) [62]. The incorporation of Sn can heal the defective sites in the MFI structure associated with silanol groups, thereby increasing the hydrophobicity of the MFI zeolite framework (Figure 5a–c). After hydrothermal treatments at 650–850 °C, the crystallinity and micropore volume of Pt–MFI samples progressively decline, and the MFI structure is nearly completely destroyed at 850 °C. In contrast, PtSn–MFI samples maintain their crystalline structure even after the treatment at 850 °C. ^29^Si solid-state NMR spectra further support this observation: a peak at −109 ppm (Q^3^ signal, originating from the hydrolysis of Si–O–Si) is evident in Pt–MFI-650-H_2_O but is absent in PtSn_10_–MFI-650-H_2_O, indicating that Sn incorporation effectively suppresses hydrolysis of Si–O–Si bonds in the MFI framework (Figure 5d–f). To investigate the stabilization effect of Sn on Pt species, the authors monitored the evolution of metal species in a dry CO + O_2_ atmosphere (CO/O_2_ = 1/5) via environmental TEM and found that, even after exposure to 1100 °C, the average Pt particle size in PtSn_5_-MFI is still well kept (~1.6 nm), demonstrating the efficacy of Sn species in protecting Pt species from severe sintering during hydrothermal treatments. Thus, the authors proposed that the healing of zeolite structural defective sites by Sn could largely enhance the zeolites’ hydrophobicity, which slows down the hydrolysis of Si–O–Si bonding and the subsequent deformation of the zeolite structure. In addition, the mobile PtO_x_ formed during high-temperature treatments (>600 °C) could also be anchored by Sn species, possibly through the Sn–O–Pt bonding interactions because of the affinity of cationic Pt species to SnO_x_ species. Following hydrothermal treatment at 650–750 °C, PtSn_5_–MFI and PtSn_10_–MFI catalysts showed only a slight increase in *T*_50_ (by approximately 10–15 °C) for CO oxidation, indicating well-preserved catalytic activity (Figure 6a,b). Although some deactivation occurred after treatment at 850 °C, the *T*_50_ values for PtSn_5_–MFI (268 °C) and PtSn_10_–MFI (249 °C) remained significantly lower than that of Pt-MFI (289 °C) (Figure 6c). The improved CO oxidation activity was attributed to the presence of Sn, which effectively stabilizes Pt nanoclusters and nanoparticles and inhibits their aggregation under high-temperature conditions. Furthermore, durability tests of PtSn_10_–MFI-850-H_2_O under harsh reaction conditions over 12 consecutive CO oxidation cycles confirmed its remarkable stability (Figure 6d).

**Figure 7:**
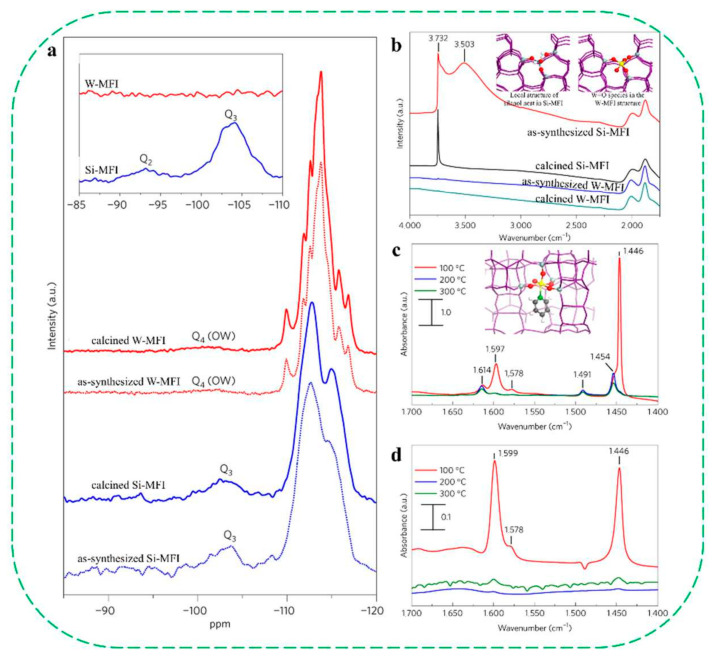
(**a**) Solid-state 29Si MAS NMR spectra of: as-synthesized Si–MFI, calcined Si–MFI, as-synthesized W–MFI and calcined W–MFI nanosized zeolite. (**b**) FT–IR spectra and local structure. FTIR spectra of as-synthesized Si–MFI, calcined Si–MFI, as-synthesized W–MFI and calcined W–MFI zeolites. Inset: Local structure of silanol nest in Si–MFI and W=O species in the W–MFI structure determined from the periodic DFT calculations. (**c**,**d**) FT–IR spectra after pyridine adsorption at 100, 200 and 300 °C. (**c**) Pyridine adsorption on W–MFI. Inset: coordination of pyridine to the W=O center in the W–MFI zeolite, according to the periodic DFT calculations. (**d**) Pyridine adsorption on Si–MFI nanosized zeolites. Reproduced with the permission of ref. [63]. Copyright 2017, Springer Nature.

In 2017, Mintova’s group reported the first synthesis of nanosized, silanol-free tungsten containing MFI zeolites (W–MFI) via a classical alkaline route [63]. The incorporation of tungsten and the absence of any silanols in the MFI structure are confirmed by ^29^Si NMR, IR, and Raman spectroscopy (Figure 7a,b). The presence of W^6+^ in the MFI framework structure was further supported by the detection of Lewis acid sites and theoretical calculations (Figure 7c,d). In the styrene epoxidation reaction, the silanol-free W–MFI exhibited the highest apparent turnover frequency (TOF = 67.9 h^−1^ after 1 h), far exceeding that of silica-supported tungsten (W-SiO_2_, TOF = 1.2 h^−1^) and W-loaded Silicalite-1 (W–Silicalite-1, TOF = 4.7 h^−1^). In addition, W-MFI demonstrated high selectivity for CO_2_ and NO_2_ removal, suggesting strong potential in environmental applications such as engine exhaust treatment and stationary gas purification.

Later, Wang et al. reported the first synthesis of W-substituted silicalite-1 with an inter-connected hollow structure (HWS-1) by post-treating the parent bulky W-substituted silicalite-1 (WS-1) through simultaneous desilication and tungstation [64]. By optimizing the NaOH/TPAOH ratio, the resultant HWS-1 retained microporosity and crystallinity while exhibiting increased enlarged mesopore volumes and external surface area compared to WS-1. The substitution of the W element could effectively saturate the defect sites in the framework and prevent the generation of silanol groups, which results in enhanced hydrophobicity in both WS-1 and HWS-1. In cyclohexene epoxidation with H_2_O_2_, HWS-1 showed superior catalytic activity compared to WS-1, which could be attributed to the HWS-1 sample with the hierarchical porosity and higher W content. Both WS-1 and HWS-1 achieved higher epoxide selectivity (>79%) than Ti-substituted Silicalite-1 (TS-1, ~50%), owing to their superior hydrophobicity and appropriately weaker acidity.

**Figure 8:**
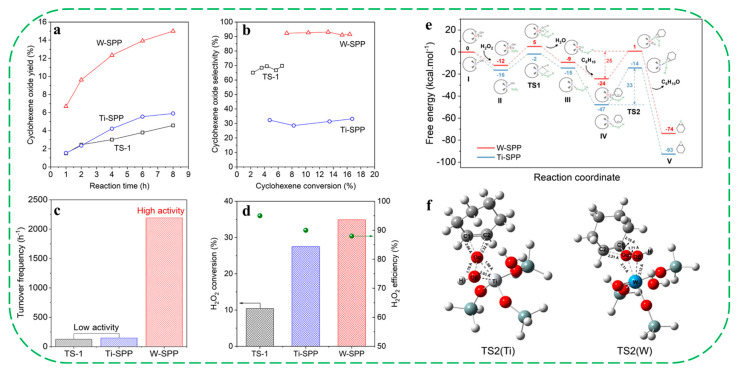
Comparison of catalytic performance in cyclohexene epoxidation over W-SPP, Ti-SPP, and TS-1: (**a**) cyclohexene oxide yield as a function of reaction time, (**b**) conversion versus selectivity, (**c**) turnover frequency, and (**d**) H_2_O_2_ consumption and efficiency (reaction conditions: 10 mmol of cyclohexene, 50 mg of catalyst, 10 mmol of H_2_O_2_ (30 wt %) as oxidant, 10 mL of CH_3_CN as solvent, 60 °C). (**e**) Energy profiles for cyclohexene epoxidation with H_2_O_2_ over cluster models of W and Ti active sites in the stepwise mechanism and (**f**) proposed transition states (TS2) of cyclohexene epoxidation over W–OOH and Ti–OOH intermediates. Reproduced with the permission of ref. [65]. Copyright 2025, American Chemical Society.

In 2025, Wang et al. reported the firstly synthesis of a well-crystalized tungsten-substituted self-pillared pentasil zeolite (W-SPP) with a house-of-card-like nanosheet morphology via one-pot hydrothermal treatment [65]. Characterization results showed that W^6+^ species were mainly coordinated in the tetrahedral framework positions as W=O segments, acting as Lewis acid sites and enhancing hydrophobicity by neutralizing silanol groups. In cyclohexene epoxidation with H_2_O_2_, W-SPP exhibited an exceptionally high TOF of 2192 h^−1^ and epoxide selectivity of 92.6%, far surpassing Ti–SPP (148 h^−1^, 28.5%) and TS-1 (125 h^−1^, 65.1%) (Figure 8a–d). FT–IR spectra of cyclohexene and cyclohexene oxide-adsorbed samples indicated that the cyclohexene adsorbed on W-SPP showed distinct bands at 3030 cm^−1^ (C-H in -CH=CH-), 1650 cm^−1^ (C=C), 1435 cm^−1^ and 1340 cm^−1^, which are all absent for Ti–SPP, suggesting that the interaction between cyclohexene and W–SPP is much stronger than that with Ti–SPP. The weak adsorption of cyclohexene oxide on W–SPP could be due to the repulsion between the epoxy group in cyclohexene oxide and W=O segments in the framework. Thus, the authors believed that the outstanding catalytic performance of W-SPP in cyclohexene epoxidation partly benefits from the active attraction of the reactant, but also the repulsion of product molecules. Theoretical calculations further elucidated that the high activity of W–SPP could be ascribed to the unique electronic structure of the H_2_O_2_-activated W active sites (W–OOH), which can distinctly reduce the activation energy barrier (25 kcal/mol) of the epoxidation step in comparison to that of Ti–OOH (33 kcal/mol). The calculation result suggests that the reactant of cyclohexene has a stronger interaction with W–SPP than with Ti–SPP, which is consistent with the above FT–IR results (Figure 8e,f). The authors believed that the high epoxide selectivity of W–SPP catalyst was further attributed to enhanced hydrophobicity, which can effectively inhibit the side-reaction of hydrolysis.

In 2003, Jaenicke and coworkers firstly synthesized a series of aluminum-free Zr–zeolite Beta catalysts with different Si/Zr ratios (i.e., 75, 100, 200) by introducing seeds from dealuminated zeolite Beta through a fluoride-assisted hydrothermal synthesis [72]. The incorporation of Zr into the framework and the well-preserved tetrahedral Si environment are ascertained by ^29^Si NMR and X-ray photoelectron spectroscopy. In the Meerwein–Ponndorf–Verley (MPV) reduction of 4-tert-butylcyclohexanone, the optimized Zr–Beta (Zr75) exhibited much higher catalytic activity (97.3%) than that of the other three catalysts, namely, Al–Beta (<0.25%), Ti–Beta (2.9%) and Sn–Beta (70.6%). The excellent catalytic performance of Zr75 could be attributed to the appropriate acidity and ligand exchangeability. Moreover, Zr–Beta (Zr100) exhibited only a 15% loss in catalytic activity under 9.1% water content in the reaction mixture, whereas Sn–Beta suffered a significant 74% decrease, indicating the superior moisture tolerance of the Zr–Beta catalyst.

Zhang et al. developed a simple one-step strategy (that is, direct grafting of Zr species on the commercial Al-Beta in ethanol) to prepare a bifunctional Zr–Al–Beta zeolite containing Lewis acidic open Zr (Ⅳ) sites and Brønsted acidic Al sites [66]. Compared to Zr–De–Al–Beta obtained from the traditional two-step strategy, Zr–Al–Beta showed less connected defects and micropore defects. The super-X-EDS mapping further confirmed that the Zr species in Zr–Al–Beta prefers to be on the surface of the crystals to form a thin Zr-rich layer. This can be attributed to the abundant silanol groups on the Al–Beta surface and is also expected to form more open Zr sites with surface terminal silanol. The ^29^Si NMR and ^1^H-^29^Si NMR spectra revealed a lower silanol concentration in Zr–Al–Beta compared to Zr–De–Al–Beta, indicating the enhanced hydrophobicity of Zr–Al–Beta. In the cascade MVP reductive etherification of cinnamaldehyde (CAL), Zr–Al–Beta achieved significantly higher CAL conversion (97.3%) and 1-cinnamyl 2-propyl ether (CPE) yield (93.9%) after 5 h than Zr–De–Al–Beta (41.0% CAL conversion and 26.9% CPE yield). The excellent catalytic performance of Zr–Al–Beta was attributed to its bifunctional activities arising from the coexistence of external open Zr (IV) sites and framework Al sites. Moreover, the catalytic activity of Zr–Al–Beta almost remained unchanged with the addition of 2 mmol of water to the reaction mixture, whereas about 50.0% decline in CAL conversion was observed for Zr–De–Al–Beta, which indicates the higher water-tolerance ability of Zr–Al–Beta related to its enhanced hydrophobicity.

**Figure 9:**
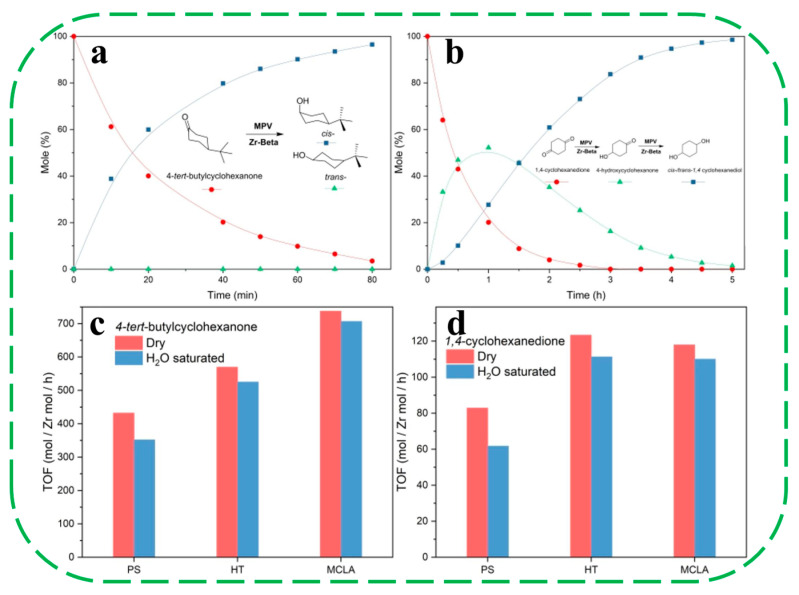
MPV reduction of (**a**) 4-tert-butylcyclohexanone (**b**) 1,4-cyclohexanedione over Zr–Beta–MCLA (Si/Zr 100) and (**c**,**d**) TOF for dry and water-saturated Zr-Beta-MCLA (Si/Zr = 100). Reaction conditions: 1.3 mmol substrate, 6.36 mL isopropanol, 82 °C, 20 mg catalyst for 4-tert-butylcyclohexanone and 100 mg catalyst for 1,4-cyclohexanedione. Reproduced with the permission of ref. [67]. Copyright 2024, Elsevier.

Recently, Zhang et al. synthesized a hydrophobic Zr–Beta zeolite with high crystallization and high metal loading, using a mechanochemistry-based liquid-assisted (MCLA) method [67]. This synthesis involved silica gel, TEAOH, NH_4_F and dealuminated Beta as seeds, with the intrinsic water in the regents facilitating good mixing during the grinding operation. The optimized MCLA-assisted synthesis conditions for Zr-Beta-MCLA were determined to be 140 °C, TEAOH/SiO_2_ = 0.30, and NH_4_F/SiO_2_ = 1.26. Compared to the fluoride-mediated hydrothermal method (15 days, Zr-Beta-HF), the MCLA method has much shorter crystallization times, allowing the rapid synthesis of Si–Beta and Zr–Beta (Si/Zr = 100) within 9 h and 15 h, respectively. EXAFS, ^29^Si NMR and TG analyses showed that Zr–Beta–MCLA featured atomically dispersed Zr in the zeolite framework without aggregation into a separate ZrO_2_ phase, an almost defect-free framework and a highly hydrophobic surface. In the MPV reduction of 4-tert-butylcyclohexanone, Zr–Beta–MCLA exhibited the highest turnover frequency (TOF = 738 h^−1^) compared with Zr–Beta–HT (TOF = 570 h^−1^) and Zr-Beta-PS (TOF = 432 h^−1^, employing post-synthesis routes) samples. As for the MPV reduction of 1,4-cyclohexanedione, the two hydrophobic catalysts, Zr–Beta–MCLA and Zr–Beta–HF, showed similar TOFs (~115 h^−1^) that were much higher than that of Zr–Beta–PS (81 h^−1^) (Figure 9a,b). After exposure to a water-saturated atmosphere at 25 °C for 4 h, the TOFs of Zr–Beta–MCLA decreased by only 4–19% in the MPV reactions, while the TOFs decreased by 8–28% and 19–70% for Zr–Beta–HT and Zr–Beta–PS, respectively (Figure 9c,d). The high crystallinity with few framework defects bestows higher hydrophobicity upon Zr–Beta–MCLA so that the presence of water has only a small impact on its catalytic activity.

**Figure 10:**
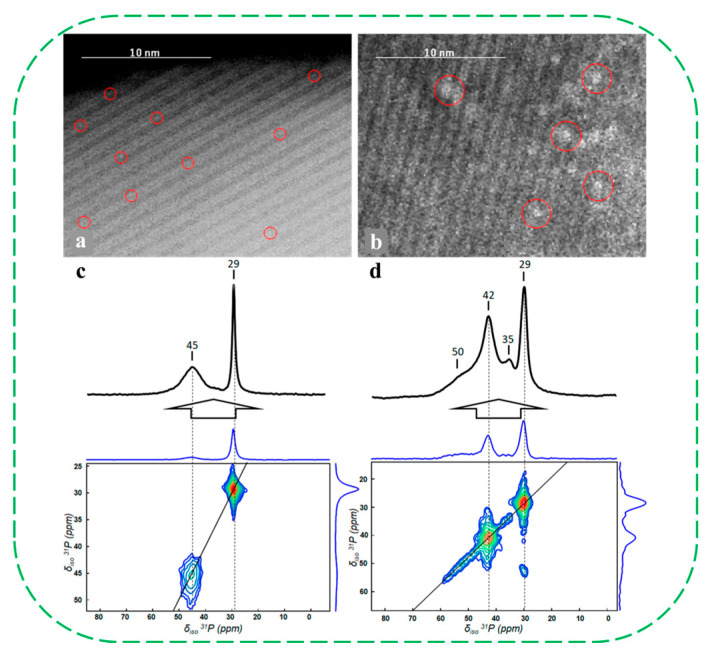
STEM-HAADF micrographs revealing the distribution of Mo species in (**a**) Mo–MFI–D and (**b**) Mo–MFI–P. ^31^P-^31^P MAS RFDR NMR spectra (top: 1D ^31^P MAS NMR spectra) of (**c**) Mo–MFI–P and (**d**) Mo–MFI–D under 1 H spinal-64 decoupling (magnetic field of 500 MHz, MAS at 12 kHz and 14 kHz for 1D and RFDR spectra, respectively). Reproduced with the permission of ref. [68]. Copyright 2019, American Chemical Society.

In 2019, Mintova’s group provided the first direct evidence of the successful incorporation of atomically dispersed molybdenum (Mo) species into the framework of nanosized MFI via hydrothermal synthesis (denoted as Mo–MFI–D) [68]. STEM-HAADF micrographs revealed that the white dot-like particles in Mo–MFI–D have a size of 0.05 nm (Mo atom) much smaller than the reference sample of Mo–MFI–P (prepared by wet-impregnation of silicalite-1). This indicates that atomically dispersed Mo atoms should be embedded in the MFI framework (Figure 10a,b). ^29^Si NMR spectra demonstrated well-resolved Q^4^ species in Mo–MFI–D, attributed to the local homogeneity of the sample. The authors proposed that Mo–MFI–D sample can be described as a silanol-free material with a high degree of local homogeneity. Using trimethylphosphine oxide (TMPO) adsorption monitored by ^31^P NMR spectroscopy, the peak at 29 ppm in Mo–MFI–D was narrower and slightly shifted (−0.5 ppm), indicating a more uniform local environment and higher hydrophobicity. In contrast, the higher chemical shift in TMPO in Mo–MFI–P suggests stronger acidity, which is attributed to the presence of abundant silanol groups and Mo oxides (Figure 10c,d).

**Figure 11:**
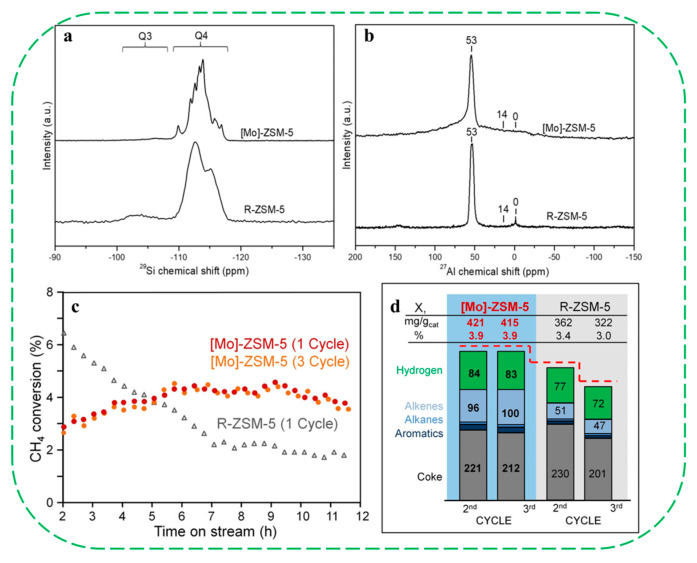
(**a**) ^29^Si MAS NMR and (**b**) ^27^Al MAS NMR spectra of samples [Mo]–ZSM-5 and R–ZSM-5. (**c**) CH_4_ conversion after the first and third consecutive cycles of reaction–regeneration as a function of time on stream for the [Mo]-ZSM-5 and reference R–ZSM-5 catalysts and (**d**) total CH_4_ conversion (X: milligrams per gram of catalyst or %), products yield (milligrams per gram of catalyst) corresponding to a 2–12 h time gap of calculation over the [Mo]-ZSM-5 and R-ZSM-5. Reaction conditions: T = 850 °C, atmospheric pressure, WHSV = 1.2 h^−1^, time on stream window = 2–12 h. Reproduced with the permission of ref. [69]. Copyright 2020, John Wiley and Sons.

In subsequent work, the same group also reported the “top-down” synthesis of novel single-site Mo-containing ZSM-5 nanozeolite with superior stability, which was obtained with the treatment of calcined nanosized aluminosilicate zeolite with sodium molybdate under autogenous pressure [69]. ^29^Si and ^27^Al MAS NMR spectra of the calcined [Mo]–ZSM-5 showed the absence of silanol groups, and only demonstrated a sharp peak at 53 ppm corresponding to Al in tetrahedral coordination (Figure 11a,b). The single-site [Mo]–ZSM-5 catalyst showed superior stability over three consecutive reaction-regeneration cycles in the high-temperature non-oxidative conversion of CH_4_, outperforming the reference sample of R–ZSM-5 prepared by the incipient wetness impregnation method (Figure 11c). Product yield analysis demonstrated that the [Mo]–ZSM-5 sample afforded a significantly higher yield of hydrogen and ethylene than the reference catalyst (Figure 11d). In particular, the [Mo]–ZSM-5 sample produces a relatively high proportion of ethylene in the C_2_ fraction (>90%). The authors proposed that the conventional R–ZSM-5 deactivates rapidly due to the sintering and migration of molybdenum carbide species as a consequence of weak interactions with the zeolites under typical multiple cycles of reaction. During the exothermic catalyst regeneration process, molybdenum carbide is oxidized, and steam is produced from the coke oxidation. Under such conditions, the Mo species become mobile and react with framework Al to form aluminum molybdates. These newly formed Mo species could not be converted back into carbides, leading to irreversible deactivation of the catalyst. In contrast, the stable performance of [Mo]–ZSM-5 over three reaction–generation cycles indicates that the catalytically active single-site Mo species within the MFI structure are well preserved. Even after undergoing regeneration cycles and steam aging, no silanol sites were detected in [Mo]–ZSM-5, indicating that the framework-incorporated Mo atoms not only stabilize the zeolite structure but also effectively prevent the formation of silanols.

**Figure 12:**
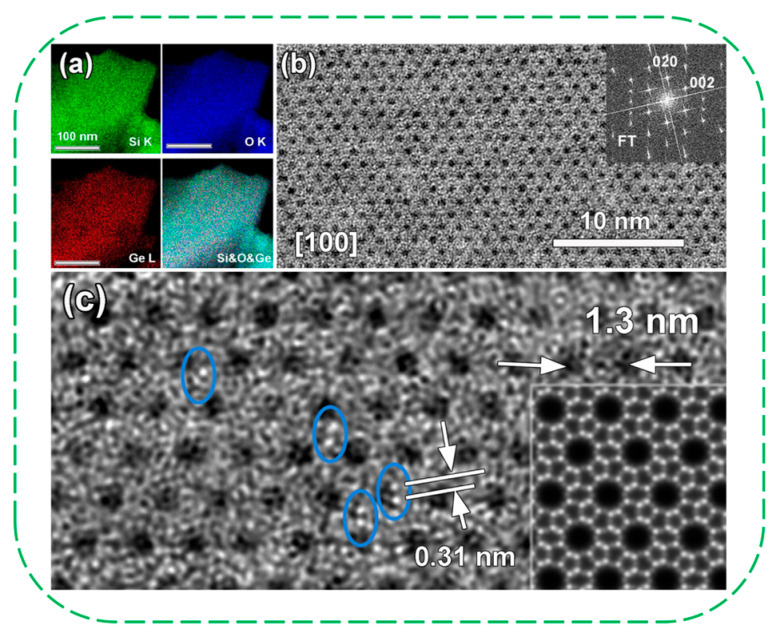
(**a**) EDX-STEM elemental maps for Si K, O K, and Ge L, along with an overlaid Si and O and Ge color image of the GeMFI sample (color coding: green—Si, blue—O, red—Ge). (**b**) High-resolution (100) HAADF-STEM image of the GeMFI sample (Inset: corresponding FT pattern). (**c**) Magnified high-resolution (100) HAADF-STEM image of the GeMFI sample, revealing the presence of Ge dimers indicated by blue circles), while not excluding the possibility of isolated Ge atoms. Inset: Simulated GeMFI image based on the monoclinic P121/n1 structure of the MFI-type zeolite, determined by XRD. Reproduced with the permission of ref. [70]. Copyright 2025, American Chemical Society.

More recently, the Mintova’s group moved their interest to the synthesis of germanium-containing zeolites with high crystallinity and thermal stability [70,73]. In 2025, they hydrothermally synthesized defect-free GeMFI zeolites containing a high germanium amount (16% Ge) in the presence of tetrapropylammonium hydroxide (TPAOH) and hydrofluoric acid [70]. Through comprehensive experimental characterization and computational modeling, they elucidated the charge compensation mechanisms responsible for the remarkable properties of these Ge-containing MFI zeolites. The charge compensation of TPA^+^ was facilitated by additional oxygen anions near the Ge atoms, primarily accommodating in the four-membered rings. The loss of resolution in the ^29^Si NMR spectra was attributed to angular and bond length disorder induced by the presence of Ge, rather than silanol defects, elucidating the exceptional hydrophobicity and stability of this zeolite at elevated temperatures. Based on core-level shift measurements, the germanium cations feature a slightly higher electron deficiency than in GeO_2_, with a coordination number exceeding the typical value of four. XAS analysis indicated that the average coordination number of Ge is predominantly five in both Ge-based samples (before and after activation), showing the high population of these double bridges between Ge dimers, without excluding the tetrahedrally coordinated Ge atoms in activated samples. High-resolution HAADF-STEM images confirmed the presence of Ge dimers, indicated by blue circles (Figure 12c). The absence of structural defects, together with the occurrence of these double bridges, explains the GeMFI zeolite with exceptional thermal and hydrothermal stability, surpassing up to 1050 °C.

In some recent works, the solvent identity and pore polarity have been demonstrated to significantly impact Lewis acid-catalyzed reactions within zeolite pores across diverse liquid-phase transformations, prompting detailed investigations into their kinetic influence [23,74,75,76]. For example, Flaherty and coauthors investigated how the polarity of microporous environments in Beta zeolite catalysts influences the TOFs of aqueous-phase sugar isomerization reactions. Their studies were conducted by compared the hydrophobic Ti–Beta–F (containing a low density of silanol defects) with the hydrophilic Ti–Beta–OH (containing a high density of silanol defects), involving the catalytic isomerization of glucose to fructose and sorbose. IR spectroscopy revealed that water forms extended hydrogen-bonding networks within hydrophilic micropores of Ti–Beta–OH, but not within the hydrophobic micropores of Beta zeolites. In Ti–Beta–OH, disruption and rearrangement of these extended hydrogen-bonded water networks at the transition state introduce entropic penalties, resulting in lower TOFs (per Lewis acidic Ti, at 373 K) compared to the low-defect Ti–Beta–F. Moreover, the higher water density in Ti–Beta–OH fosters the second solvation shells around the transition state, restricting conformational freedom and entropically destabilizing the reaction pathway. The intraporous silanol defect density thus influences the confined solvent structures present during catalysis and, in turn, dictates the stability of intermediates and transition states that control reactivity. These findings underscore the need for synthetic approaches capable of precisely controlling defect density to enable post-synthetic routes for preparing metal zeolites with predictable and targeted catalytic properties [74].

Later, Gounder and coauthors systematically investigated how intraporous hydrophilic silanol defects influence the sugar isomerization of Sn-Beta zeolites, and developed a suite of quantitative methods to assess defect densities (intraporous silanol groups), including methanol-packing density measurements and CD_3_CN infrared spectroscopy. Their findings reveal that densely distributed silanol groups within micropores can stabilize hydrogen-bonded water networks during catalysis that require disruption and rearrangement at isomerization transition states to incur entropic penalties, finally leading to the decrease in catalytic reaction rates. In contrast, dilute intraporous silanol densities (low-defect analogs) are unable to stabilize extended water networks, resulting in higher reactivity. To achieve catalytic performance comparable to that of hydrophobic Lewis acid zeolites (e.g., hydrothermally synthesized Sn–Beta) synthesized via hydrothermal methods, the authors proposed that the density of intraporous defect sites in post-synthetically prepared Lewis acid zeolites must be reduced below the critical threshold values required to sustain extended hydrogen-bonded water networks [75].

**Figure 13:**
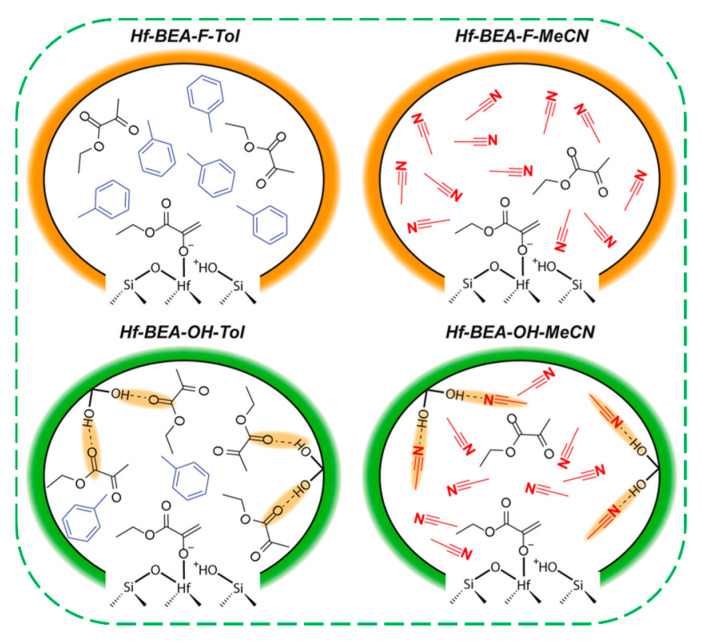
Depictions of pore environments in Hf–BEA–F–Tol, Hf–BEA–F–MeCN, Hf–BEA–OH–Tol and Hf–BEA–OH–MeCN, based on kinetic and EP absorption data. Reproduced with the permission of ref. [23]. Copyright 2023, American Chemical Society.

Recently, Khechfe et al. synthesized two Hf–Beta zeolites with distinct framework polarities: hydrophobic Hf–BEA–F (Si/Hf = 327) via fluoride-mediated hydrothermal synthesis, and hydrophilic Hf–BEA–OH (Si/Hf = 145) by grafting Hf into the dealuminated BEA sample [23]. They examined the effects of framework polarity and solvent identity on the self-aldol addition rates of ethyl pyruvate (EP), a model biomass-derived compound, catalyzed by two Hf-Beta zeolites in toluene and acetonitrile. The reaction exhibited first-order kinetics across the entire EP activity range (0.02–0.40) for all four catalytic systems, consistent with the nucleophilic enolate attack as the rate-determining step and a single adsorbed EP as the predominant reactive intermediate (Figure 13). At 363 K, the apparent first-order rate constants (k_app_) across the four systems vary by up to two orders of magnitude. The highest reaction rate is observed for the hydrophobic Hf–BEA–F in toluene (k_app_ = 0.36 (mmol) (mmol closed Hf)^−1^(s)^−1^), while the lowest is recorded for the hydrophilic Hf–BEA–OH in acetonitrile (k_app_ = 0.0026 (mmol) (mmol closed Hf)^−1^(s)^−1^). Apparent enthalpic and entropic barriers in hydrophobic Hf–BEA–F are within the experimental error of each other for both toluene and acetonitrile, despite rate constants that are four times lower in acetonitrile. In contrast, a pronounced solvent effect is observed in the hydrophilic Hf–BEA–OH, where the apparent enthalpic in Hf-BEA-OH-Tol is approximately 15 kJ/mol higher in Hf–BEA–OH–Tol than in the other three systems. Adsorption isotherm measurements of EP on Si–BEA–F and Si–BEA–OH reveal that in acetonitrile, EP uptake is negligible regardless of the framework polarity, suggesting that the pores are fully saturated with the solvent. In contrast, EP uptake increases with activity in toluene, indicating the presence of a mixture of the solvent and EP environment within the pores. Their findings reveal that in hydrophobic Hf–BEA–F, polar solvents have minimal impact on substrate adsorption kinetics. However, in hydrophilic Hf–BEA–OH, the silanol nests can hinder EP adsorption via hydrogen bonding. In both catalysts, polar solvents primarily reduce aldol reaction rate constants through active site poisoning, which is less pronounced in hydrophobic frameworks.

The above results demonstrated that metallosilicate zeolites with tunable Lewis acidity, potential redox activity and high surface hydrophobicity have shown some advantages in various aqueous or water-containing reaction systems (e.g., alkene epoxidation, sugar isomerization and Baeyer–Villiger oxidation). The enhanced hydrophobicity could effectively suppress competitive water adsorption on the catalytically active sites, thus being beneficial for the improvement of the catalytic efficiency and the stability of the zeolite-based catalysts. In addition, incorporating other noble metals (e.g., Pt, Au) or constructing hierarchical pore structures may significantly enhance the catalytic performance of the metallosilicate-based zeolites. Currently, the effective utilization of Ti-containing metallosilicates (e.g., TS-1) has been conducted in industrial production of olefin epoxides, revealing the great potential for further commercial application of the hydrophobic metallosilicate-based catalysts. However, more concern should be given to the synthesis of the metallosilicate zeolites, since various metal species such as non-framework metal or metal oxide clusters are easily formed during the synthesis of metallosilicates, which are commonly caused by the difference in hydrolysis/condensation rates of the metal precursors and the silicon source. Such mismatch may have some negative effects on the physicochemical properties of the metallosilicate zeolites, including hydrophobicity/hydrophilicity, and finally leading to an adverse impact on their catalytic performance. Therefore, future research efforts should mainly focus on developing more simple and effective synthesis methods to obtain metallosilicate zeolites with a uniform dispersion of metal species and desirable hydrophobicity for the target reaction. In addition, exploring new types of metallosilicate zeolite catalysts to adapt to different catalytic reactions is also an important direction for future research. 

## 3. Hydrophobic Modification of Zeolites

The modification of zeolites is an effective approach to enhancing hydrophobicity, primarily by removing and/or healing structural silanol defects [11,46,77]. Among these approaches, some of the most promising and recurring methodologies on defect healing, including liquid-mediated treatments, silanization modification and direct hydrothermal synthesis, will be addressed below.

### 3.1. Liquid-Mediated Post-Synthetic Modification

The liquid-mediated modification generally refers to the use of fluoride species (e.g., HF and NH_4_F), in the presence of pore-filling hydroxide (e.g., tetraethylammonium hydroxide), to induce silicate migration, thereby promoting framework reconstruction and defect healing in the zeolite [46,77,78,79,80,81]. In contrast to conventional silanization modification, which frequently leads to micropore volume shrinkage or pore blockage of the framework (attributed to the condensed silylating agents), the liquid-mediated modification may completely avoid these issues and has been widely applied to heal defects in high-silica Beta, ZSM-5, and MOR zeolites, exhibiting excellent hydrophobicity and hydrothermal stability [78,81].

**Figure 14:**
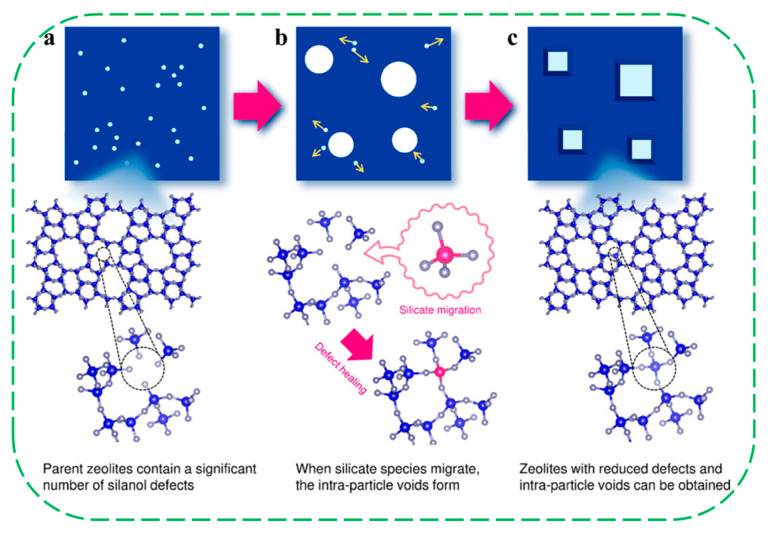
Proposed mechanism for self-defect healing. (**a**) parent zeolites contain a significant number of silanol defects. (**b**) when silicate species migrate the intra-particle voids form. (**c**) zeolites with reduced defects and intra-particle voids can be obtained. Reproduced with the permission of ref. [78]. Copyright 2020, American Chemical Society.

In 2020, Wakihara and coworkers proposed a method for healing framework defects to create extremely stable and highly hydrophobic high-silica zeolites. A series of high-silica (SiO_2_/Al_2_O_3_ > 240) zeolites, like *BEA-, MFI, and MOR-type topologies, could be stabilized by greatly reducing the number of defect sites through a liquid-mediated treatment without using additional silylating agents [78]. This treatment employed a fluoride anion (NH_4_F) and pore-filler cation (TEAOH). The treated zeolites could withstand extremely high-temperature steaming conditions at 900–1150 °C and retain crystallinity and micropore volume, whereas the parent commercial zeolites degraded completely. Furthermore, the reduction in defect density endowed the treated materials with enhanced hydrophobicity. The authors proposed a self-defect-healing mechanism to decrease the number of defect sites and form intraparticle voids. The parent zeolites contain a significant number of silanol defects that are invisible by microscopy (Figure 14a). The silicate species migrate from somewhere in the framework and can heal defects through condensation. During this process, the defects also move and form intraparticle voids via the migration of silicate species. All of these silicate species originate from the same framework, and some defects are transported to the crystal edges (Figure 14b). After the treatment, the remaining crystal structure contains a drastically reduced number of defects (Figure 14c). However, the self-defect-healing approach is not suitable for some zeolites with lower Si/Al ratio. They proposed that the different behavior of high- and low-silica zeolites likely arises from distinct degradation mechanisms. In high-silica zeolites, silanol defect sites primarily influence the stability of zeolites, whereas in low-silica zeolites, dealumination plays the dominant role. Even if the defect sites are healed, dealumination can readily occur during high-temperature steaming, leading to structure degradation.

**Figure 15:**
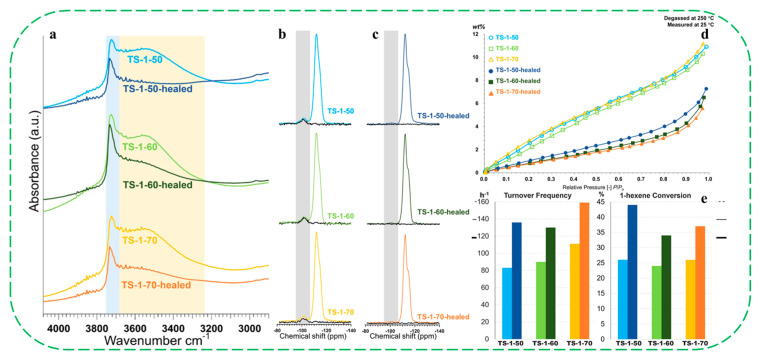
(**a**) Normalized FT-IR plots of the TS-1 and TS-1-healed samples. (**b**,**c**) Spectra of the ^29^Si single-pulse MAS NMR (colorful) and CP MAS NMR (black) of (**b**) TS-1 and (**c**) TS-1-healed samples. (**d**) Water vapor adsorption isotherms of TS-1 and TS-1-healed samples. (**e**) Comparisons of catalytic performance of pristine TS-1 (lighter colors) and defect healing-treated TS-1 (darker colors). Reproduced with the permission of ref. [80]. Copyright 2023, American Chemical Society.

Later, the same group reported the successful elimination of silanol defects in TS-1 zeolites, yielding materials with a highly hydrophobic nature [80]. After defect-healing treatments, all samples showed negligible changes in crystallinity, morphology, particle size, and Ti coordination and content. Characterization by FT-IR, ^29^Si MAS NMR and water vapor adsorption isotherms confirmed the complete removal of silanol defects and a significantly improved hydrophobicity (Figure 15a–d). The defect-healed TS-1 zeolites exhibited highly enhanced catalytic performance in the epoxidation of 1-hexene with H_2_O_2_, with 1-hexene conversion increasing by up to 70% relative to the pristine samples. A turnover frequency as high as 159 h^−1^ was achieved after defect healing (Figure 15e). Catalytic tests of alkene epoxidation revealed significantly performance enhancements in defect-healed TS-1 catalysts, attributed to the elimination of silanol defects and the reduced number of isolated Si–OH groups. The authors believed that silanol defects are one of the most detrimental factors affecting alkene oxidation catalysis over TS-1 catalysts when using aqueous H_2_O_2_. This study provides an effective strategy for synthesizing silanol defect-free TS-1, which provides new opportunities to understand fundamental principles of Ti-based catalyst improvement via hydrophobicity manipulation.

It should be noted that although the conventional techniques (e.g., ^29^Si MAS NMR and FT-IR) are highly informative for assessing the average silanol density and the overall hydrophobicity of zeolites, they possess inherent limitations related to detecting subsurface defects or capturing nanoscale spatial heterogeneity in hydrophobicity. To fully determine the hydrophobicity distribution of zeolite-based catalysts, particularly around the catalytically active sites, advanced characterization methods such as ultrafast cameras and magnetic resonance imaging (MRI), in situ liquid atomic force microscopy (AFM) and neutron scattering could be conducted to offer more valuable insights. These techniques are capable of probing local hydrophobic environments and water organization at the solid–liquid interface with sub-nanometer resolution, thereby providing a more dynamic and spatially resolved picture of hydrophobicity, which can be more useful to correlate it directly with their catalytic function [4,82].

The above research results showed that the fluoride-mediated post-synthesis modification strategy represents one of the most effective approaches for enhancing the hydrothermal stability and hydrophobicity of zeolites. This method is primarily employed to heal structural defects in high-silica zeolites by eliminating silanol groups in the framework, demonstrating considerable potential in applications such as epoxidation reactions and CO_2_ capture. However, this strategy also faces several problems. First, the high cost of high-purity fluoride reagents, combined with the frequent need for excess usage during modification, significantly increases the synthesis costs. Second, due to the highly corrosive and toxic nature of fluorides, strict requirements are imposed on reaction equipment (e.g., corrosion-resistant reactors) and operational protocols (e.g., personal protection and waste disposal). Furthermore, the relatively harsh synthesis conditions and the need for precise control over crystal defects pose difficulties for large-scale reproducible preparation. Although this strategy may currently be difficult to directly apply in the large-scale production of bulk chemicals, the resulting catalysts exhibit exceptional properties, such as ultra-high hydrophobicity and a defect-free framework structure, making them highly valuable for high-value-added applications, such as the synthesis of important pharmaceutical intermediates, where higher catalytic activity and selectivity can offset the elevated production costs. Future research should focus on exploring new strategies such as fluoride recycling or developing milder and safer fluoride-free reagents to improve the practicality and economic viability of this method. In addition, it is also essential to systematically evaluate the environmental footprints of these alternatives using life-cycle analysis (LCA).

### 3.2. Silanization Post-Modification of Zeolites

Silanization modification usually refers to a grafting reaction between the silylation reagent and the hydroxyl groups (Si–OH) on the zeolite surface, which could block the hydrophilic surface hydroxyl groups by forming hydrophobic Si–O–Si–R_3_ bonds, thus improving its hydrophobicity while maintaining other physicochemical properties, including acidity/redox properties [83,84,85,86]. Silylation agents usually include dodecyltriethoxysilane, triethoxyfluorosilane, trimethylchlorosilane, octadecyltrichlorosilane and so on. To date, it has been reported in a number of studies that the silanization of aluminosilicate and metal-encapsulated zeolites could effectively modulate the hydrophobic environment of the zeolites, thereby enhancing their catalytic performance [34,85,87,88,89,90,91,92].

Recently, Wang et al. synthesized a series of nanosized Beta zeolites with different Si/Al ratios (denoted as Nano–H–Beta-*n*) via two-stage varying temperature crystallization of a concentrated gel containing a small amount of _L_-lysine additives [87]. They observed that product selectivity was closely related with the Si/Al ratio in the alkylation of furan (FUR) and furfural alcohol (FA) reaction. The higher Si/Al ratios favored the selective formation of bis (furan-2-yl) methane (BFM). The condition-optimized Nano–H–Beta-180 exhibited superior efficiency, delivering an initial turnover frequency (TOF) of 433 h^−1^ and a BFM yield of 91%, outperforming commercial H–Beta-180–C with the same Si/Al ratio (TOF = 166 h^−1^, BFM yield = 79%). In order to further improve the catalytic efficiency, Nano–H–Beta-180 was functionalized with triethoxy fluorosilanes (TEFS) to enhance hydrophobicity. The resulting TEFS-functionalized and pyridine-protected sample (Nano-H–Beta-180–Py–TEFS) achieved a greatly higher BFM yield (97%) and selectivity (98%) compared with the unmodified Nano–H–Beta-180. The enhanced hydrophobicity also exhibited improved stability and water resistance relative to its pristine counterpart. Kinetic analysis revealed that the apparent activation energy (E_a_) for BFM synthesis over Nano-H–Beta-180–Py–TEFS was 36.3 kJ/mol, significantly lower than that of Nano–H–Beta-180 (57.4 kJ/mol), indicating a shift in the rate-limiting step and a reduction in the kinetic barrier for carbocation intermediate formation. Additionally, Nano–H–Beta-180–Py–TEFS exhibited enhanced FA activation, as reflected by a substantially lower apparent reaction order with respect to FA compared to the unmodified catalyst. Under more demanding continuous-flow conditions, the Nano–H–Beta-180–Py–TEFS catalyst maintained nearly 100% activity and selectivity for 200 h on stream, achieving a turnover number (TON) of 197, which is considerably higher than that typically obtained in batch reactions (TON = 98). These results suggest that the continuous-flow alkylation process employing Nano-H–Beta-180–Py–TEFS offers a promising route toward more efficient and safer targeted transformations of FUR into BFM.

In 2023, Martins and coworkers reported the synthesis of two MWW-topology zeolites, namely microporous MCM-22 and hierarchical ITQ-2, which were functionalized with dodecyltriethoxysilane and evaluated in a two-phase glycerol ketalization reaction with acetone [85]. Contact angle measurement revealed that the hydrophobicity of the functionalized samples (MCM-22F and ITQ-2F) increased compared with their parent counterparts. Catalytic tests showed that ITQ-2 exhibited a TOF_0_ (turnover frequency at time zero) two times higher than MCM-22, attributed to improved accessibility. By examining the relationship between TOF and the glycerol contact angle, the authors found that hierarchical ITQ-2 displayed a fitted slope that was twice that of microporous MCM-22, indicating that hydrophobicity played a more significant role in ITQ-2. Both the functionalized MCM-22F and ITQ-2F presented higher TOF_0_ than the parent MCM-22 and ITQ-2 catalysts, which could be mainly attributed to enhanced hydrophobicity. This improvement prevented water solvation of acid sites, allowing catalytic reactions to occur more effectively at the phase boundary. The above results verified that ITQ-2F was the most efficient catalyst, benefiting from both its contributions from the hierarchical structure and the attenuation of water interaction due to functionalization.

**Figure 16:**
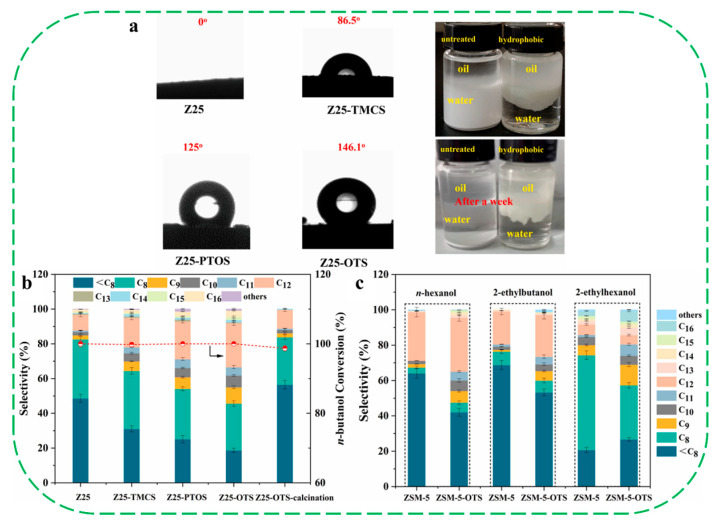
(**a**) The water contact angle and the dispersion behavior in mixture of water and oil for different catalysts. (**b**) Product distribution of *n*-butanol dehydration-oligomerization over different catalysts. (**c**) Production of jet-fuel from different higher alcohols. Reaction conditions: alcohols, 10 g; catalyst, 1 g; reaction time, 12 h; reaction temperature, 260 °C. Reproduced with the permission of ref. [34]. Copyright 2025, Elsevier.

More recently, Liu et al. reported the preparation of a series of hydrophobic HZSM-5 zeolites, constructed by the modification of chlorosilanes with different chain lengths, including trimethylchlorosilane (TMCS), propyltrichlorosilane (PTOS), and octadecyltrichlorosilane (OTS) [34]. With increasing chlorosilane chain length, the water contact angle increased from 0° for unmodified Z25 to 146.1° for Z25–OTS, indicating a progressive enhancement in hydrophobicity. Dispersion experiments in a water–oil mixture revealed that Z25 preferentially dispersed in the aqueous phase, whereas Z25–TMCS, Z25–PTOS, and Z25–OTS dispersed in the oil phase (Figure 16a). In the dehydration-oligomerization of *n*-butanol, the three hydrophobic catalysts (Z25–TMCS, Z25–PTOS and Z25–OTS) achieved significantly higher C_8_-C_16_ selectivity (>68.7%) compared with the hydrophilic Z25 (51.3%) (Figure 16b). Among them, Z25–OTS exhibited an unprecedented C_8_-C_16_ selectivity of 80.5%, accompanied by a marked decrease in C_8_ content and a substantial increase in C_12_ content from 9.1% (Z25) to 25.3% (Z25–OTS). In the dehydration-oligomerization of other higher alcohols derived from ethanol upgrading (*n*-hexanol, 2-ethylbutanol, 2-ethylhexanol), Z25–OTS also exhibited better dimerization of these higher alcohols compared with the parent Z25 (Figure 16c). When *n*-hexanol was utilized as the reactant, Z25–OTS showed much higher selectivity of C_8_-C_16_ fraction (58.2%) and C_12_ fraction (30.5%) than that of parent Z25 (C_8_-C_16_ of fraction 35.6%, C_12_ fraction of 27.5%). The exceptional performance of Z25–OTS was attributed to its enhanced hydrophobicity, higher Brønsted/Lewis acidity, and increased hydrocarbon adsorption capacity. By combining a series of characterization results, a deep mechanistic understanding of the relationship between the zeolite hydrophobicity and the C_8_-C_16_ selectivity has been proposed. Firstly, hydrophobic modification alters the diffusion behavior of water and hydrocarbon molecules: water molecules are hindered from entering the zeolite pores, whereas hydrocarbon molecules undergo bidirectional diffusion along the hydrophobic surface. Due to the affinity between hydrophobic alkyl chains and the modified surface, hydrocarbons tend to accumulate on the surface of zeolite, promoting coupling reactions of olefin intermediates. Second, the results of *n*-butene–TPD revealed that the stronger adsorption of hydrocarbon species on hydrophobic zeolites, combined with delayed desorption, further facilitates the formation of long-chain hydrocarbon products.

### 3.3. Direct Hydrothermal Synthesis of Hydrophobic Zeolite-Based Catalysts

Compared with the post-modification method, direct hydrothermal synthesis/modification of hydrophobic zeolites seems more attractive since it might achieve a much more uniform distribution of hydrophobic organic groups over the zeolites. In the work reported by Ryoo and coauthors, they successfully synthesized hierarchical MFI (or BEA) zeolites with specific morphology (pillared nanosheets or rod-like crystals) using a Gemini-type piperidine-based multi-ammonium surfactant (N6-diphe) [82,93,94]. On the basis of a variety of characterization results, they proposed that the smaller mesopores (6–8 nm) that existed in the hierarchical zeolite should originate from the hydrophobic alkyl chains, while the larger mesopores (10–30 nm) are related to the spinodal decomposition-type segregation of phases consisting of as-synthesized hydrophobic zeolites and the remaining water [82]. These results suggest that the usage of Gemini-type template like N6-diphe is highly effective for the direct self-assembly synthesis of hydrophobic zeolites with special morphology and hierarchical structure, which may have promising potential for the applications in heterogeneous catalysis (e.g., macromolecule conversion and biomass catalysis) and adsorption/separation processes (e.g., VOC removal and CO_2_ capture).

Recently, Li and coauthors synthesized Beta zeolites with hydrophobic pores, using a template-free method combined with in situ silicon-source modification via tetraethoxyfluorosilane (TEFS) [88]. The TEFS-mediated synthesis increased both the total acid amount and the strength of strong acid sites (especially the Brønsted acid sites) while also improving pore hydrophobicity. Compared with conventional Beta zeolite, the TEFS-mediated Beta samples showed higher lactic acid conversion (96% vs. 85%) and lactide yields (84% vs. 70%), which was attributed to the increase in Brønsted acid sites and the improvement of the pore hydrophobicity. The authors proposed that the unique hydrophobic property can rapidly remove water molecules near the active sites in zeolites, promoting the esterification reaction to proceed in the forward direction. Meanwhile, the rapid removal of water could prevent the hydrolysis reaction between lactide and water molecules under the catalysis of Brønsted acid, thereby increasing the yield of lactide.

In 2018, Xiao and coauthors developed a core–shell catalyst, TiO_2_@HP-zeolite, by fixing TiO_2_ particles within the hydrophobic zeolite (HP-zeolite) crystals [89]. The HP-zeolites were synthesized via in situ functionalization of the faujasite framework with organic groups (e.g., -CH_3_, -Ph, and -CF_3_) during hydrothermal crystallization. Among the catalysts tested, the hydrophobic Y zeolite fixed TiO_2_ (TiO_2_@Y-Me-15) exhibited superior performances in the complete removal of wet formaldehyde under continuous irradiation (λ > 350 nm) in a long period, outperforming the physical mixture of TiO_2_ and Y-Me-15, conventional Y zeolite fixed TiO_2_ (TiO_2_@Y), and pure TiO_2_ catalysts. This outstanding performance was attributed to the core–shell architecture, which effectively anchored TiO_2_ within the HP-zeolite crystal, thereby maximizing the synergistic effect between the wettability-selective hydrophobic sheath and the photocatalytically active TiO_2_ particles.

In another work reported by the same group, they designed a catalyst featuring a “molecular fence” to effectively enrich H_2_O_2_ in situ during the methane oxidation to methanol reaction [90]. The catalyst was synthesized by fixing AuPd alloy nanoparticles within ZSM-5 zeolite crystals (denoted as AuPd@ZSM5), followed by modifying the external surface of the zeolite with organsilanes (denoted as AuPd@ZSM-5-C_16_). The hydrophobic sheath acted as a molecular fence, permitting hydrophobic CH_4_ molecules to enter the zeolite pores while preventing the in situ-formed hydrophilic H_2_O_2_ from diffusing away. This configuration provided the optimal combination of high local peroxide concentration and rapid methane adsorption, thereby enhancing its catalytic performance. As a result, the methanol selectivity reached 92% at a CH_4_ conversion of 17.3%, with a methanol productivity of 645.1 mmol g_AuPd_^−1^ h^−1^. In contrast, the AuPd@ZSM-5 catalyst without hydrophobic shells achieved only 6.3% CH_4_ conversion and a methanol productivity of 210.9 mmol g_AuPd_^−1^ h^−1^.

Recently, Tian et al. reported the hydrothermal synthesis of a novel hollow Pt@O-ZSM-5@OTS catalyst designed to protect Pt nanoparticles [91]. In this structure, Pt was integrated into hollow ZSM-5 prepared by a dissolution–crystallization strategy and subsequently coated with hydrophobic octadecyltrichlorosilane (OTS), functioning like a waterproof carapace. In the unique hollow structure, the crystalline framework, porosity, and active Pt sites of Pt@O-ZSM-5@OTS were well maintained as the OTS coating amount was tuned, while the external surface was converted from hydrophilic to hydrophobic. The optimized Pt@O-ZSM-5@OTS2 demonstrated superior catalytic activity for benzene oxidation and remarkable stability in environments containing 10 vol% H_2_O for 120 h, whereas the benzene conversion over uncoated Pt@O-ZSM-5 dropped from 90% to 50%. Finite element modeling, competitive adsorption experiments, and DFT simulations revealed that the synergy between the hollow structure and OTS coating created a natural protective barrier around the confined Pt nanoparticles. This barrier hindered moisture penetration into the cavity, while promoting the preferential diffusion and oxidative degradation of benzene under high-humidity conditions.

**Figure 17:**
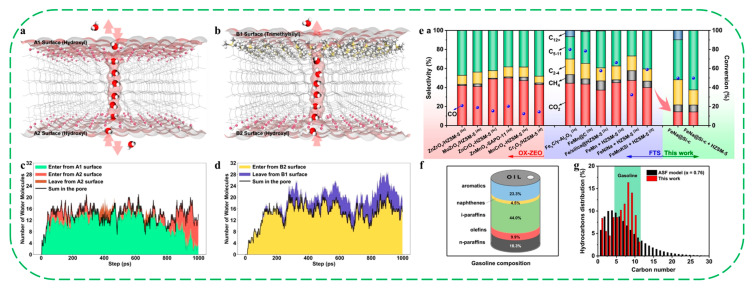
Molecular dynamics simulations. Diffusion model of water molecules through (**a**) structure A and (**b**) structure B. The number of water molecules entering/leaving the pore from different surfaces of (**c**) structure A and (**d**) structure B. “Sum in the pore” means the number of water molecules existing in the pore. Catalytic performance for syngas to gasoline. (**e**) Product’s distribution of FeNa@Si-c+HZSM-5 in comparison with the previous works. (**f**) Gasoline composition obtained over FeNa@Si-c+HZSM-5. (**g**) Hydrocarbons distribution compared with the ASF model. Reproduced with the permission of ref. [95]. Copyright 2023, John Wiley and Sons.

In 2023, Xu et al. designed a bifunctional catalyst consisting of hydrophobic FeNa@Si-c and HZSM-5 zeolite for the conversion of syngas to gasoline [95]. First, hydrophobicity was achieved by introducing nonpolar chlorotrimethylsilane through a silanization reaction, yielding the FeNa@Si-c catalyst. The hydrophobic FeNa@Si-c was then combined with nanosized HZSM-5 zeolite to create a bifunctional system containing iron carbide and acid sites. At a CO conversion of approximately 50%, the CO_2_ selectivity of FeNa@Si+HZSM-5 catalyst (FeNa@Si without -CH_3_ groups) was 2.1 times higher than that of FeNa@Si-c+HZSM-5. Moreover, calcining FeNa@Si-c in air could remove the -CH_3_ groups, rendering the catalyst hydrophilic and increasing its CO_2_ selectivity to levels similar to FeNa@Si. These results suggest that the enhanced hydrophobicity of the catalyst was important to inhibit CO_2_ formation during syngas to gasoline. Molecular dynamic simulations and model experiments revealed that the water diffusion in the hydrophilic catalyst was bidirectional, whereas in the hydrophobic catalyst it was unidirectional, which was crucial to tune the water–gas shift reaction and control CO_2_ formation (Figure 17a–d). The hydrophobic FeNa@Si-c+HZSM-5 bifunctional catalyst achieved a high selectivity of C_5_-C_11_ (62.5%) with a CO conversion of 49.8% in the conversion of syngas to gasoline reaction. Importantly, the CO_2_ selectivity was only 14.3%, much lower than that of the oxide–zeolite (OX–ZEO) and traditional Fe–Fischer–Tropsch synthesis (Fe–FTS) catalysts (Figure 17e). In the produced gasoline, the content of high-octane components (aromatics, olefins, naphthenes, and isoparaffins) reached 81.7%. Moreover, the proportions of aromatics and olefins were controlled at 23.3% and 9.9%, respectively, which is significantly lower than those reported in the previous works (Figure 17f). The above results indicate that the FeNa@Si-c+HZSM-5 bifunctional catalyst was highly efficient for synthesizing high-quality gasoline from syngas, and it exhibited a strong ability to suppress the formation of the undesired CO_2_ byproduct.

**Figure 18:**
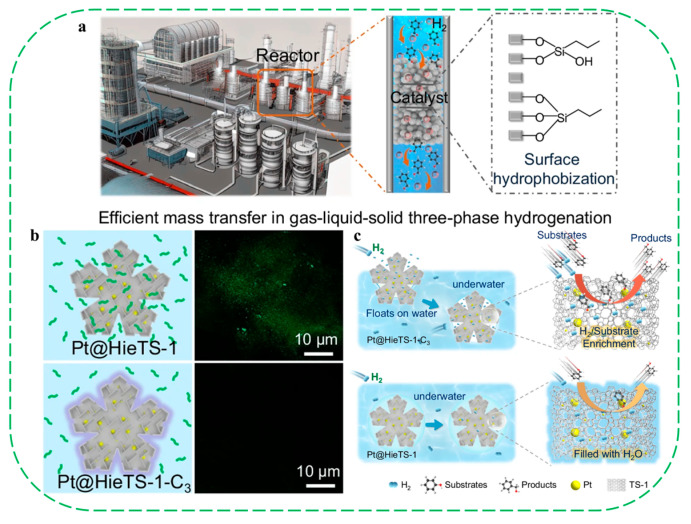
(**a**) Schematic representation of zeolite nanoreactor catalyst in gas–solid three phase hydrogenation. (**b**) Schematic diagram of Pt@HieTS-1 and Pt@HieTS-1-C_3_ immersed in water containing fluorescent dye and CLSM images of Pt@HieTS-1 and Pt@HieTS-1-C_3_. (**c**) Schematic illustrations of the hydrogenation reaction occurring on Pt@HieTS-1-C_3_ and Pt@HieTS-1. Reproduced with the permission of ref. [92]. Copyright 2024, Springer Nature.

Similarly, Wang et al. reported a nanoreactor (Pt@HieTS-1-C_x_) featuring joint gas–liquid interfaces and controlled wettability for boosting H_2_ gas and substrates to involve reactions (Figure 18a) [92]. The catalyst was synthesized by fixing Pt active sites on a hierarchical TS-1 zeolite via a ligand-protected hydrothermal approach, followed by hydrophobic modification of the external surface through silylation with organosilanes. Characterization by XRD, N_2_ adsorption–desorption isotherm, XPS, FT-IR and water contact angle measurements confirmed that the introduced alkyl groups impart high hydrophobicity without significantly affecting the zeolite pore structure or the chemical state of Pt active sites. The hydrophobicity at the micro-nanoscale was further investigated using confocal laser scanning microscopy (CLSM) with fluorescein sodium as a water-soluble fluorescent probe. Since fluorescence occurs only in water-accessible regions, Pt@HieTS-1 exhibited strong fluorescence throughout its body, whereas Pt@HieTS-1-C_3_ showed only weak signals, indicating that water could not infiltrate its micro-mesoporous channels (Figure 18b). In aldehyde/ketone hydrogenation, Pt@HieTS-1-C_3_ achieved a reactivity 4.3 times higher than Pt@HieTS-1, with a turnover frequency (TOF) of 92.3 h^−1^, 3.2 times greater than the unmodified catalyst (Pt@HieTS-1). The exceptional performance of Pt@HieTS-1-C_3_ was attributed to the hydrophobic/aerophilic nature of the silane sheath, which allowed H_2_ molecules to diffuse through the zeolite channels with minimal resistance and directly access Pt active sites from the gas phase, significantly increasing local H_2_ concentration. Meanwhile, the lipophilic substrate molecules exhibited a strong affinity for the silane sheath, promoting their enrichment around Pt sites. After hydrogenation, the hydrophilic alcohol products rapidly diffused into the aqueous solvent because of their similar polarity (Figure 18c).

The above research progress demonstrates that it is an effective approach to enhance the hydrophobicity of zeolites by post or direct modification with silylating agents containing organic groups. This process significantly improves hydrophobicity and compatibility with organic reactants, protects acid sites from water-induced deactivation, thus enhancing their catalytic performance in various reactions, including the conversion of lactic acid to lactide, two-phase glycerol ketalization, ethanol dehydration-oligomerization to higher alcohols, the alkylation of furan and furfuryl alcohol, and the conversion of syngas to high-valued products. Currently, research on introducing small amounts of hydrophobic templates during zeolite synthesis to directly obtain hydrophobic zeolites remains relatively limited, which may be attributed to the poor stability of such templates under high-temperature calcination conditions. In addition, there is still a lack of cost-effective, well-established hydrophobic templates with excellent thermal stability. In contrast, post-synthetic silanization modification strategies are more widely adopted. However, this approach faces still several problems, such as the toxicity of certain silane reagents (e.g., fluorosilanes and chlorosilanes), instability of the grafted silane layers (which are prone to hydrolysis or thermal degradation at elevated temperatures), and the risk of pore blockage (non-selective deposition of silylating agents). Future efforts should focus on developing more green and efficient silylating agents (e.g., bio-based agents) to advance the sustainable optimization and practical application of the hydrophobic zeolite-based catalysts.

## 4. Constructing Hydrophobic Zeolite-Based Composites

Another attractive approach for improving the hydrophobicity of zeolite-based catalysts is to coat the hydrophilic zeolite crystals or metal-supported zeolite catalysts with a thin layer of hydrophobic pure-silica zeolite, which can generate core–shell structured composites with enhanced water tolerance [96,97]. For instance, silicalite-1, a pure-silica zeolite with MFI topology, is becoming an ideal hydrophobic surface-coating material that can reduce the density of external acid sites, protect catalytically active sites (e.g., metal nanoparticles), and enhance the hydrothermal stability of zeolite-based catalysts.

In an early work reported by Qu and coworkers, a series of core–shell zeolites (denoted as CS_1_, CS_2_, CS_3_) comprising Fe-ZSM-5 as core and silicalite-1 as shell (with different thickness of silicalite-1 shell) were synthesized via secondary hydrothermal crystallization [98]. During the secondary crystallization process, a small amount of Fe^3+^ and Al^3+^ was leached from the Fe–ZSM-5 framework along with partial dissolution of the Fe-ZSM-5 core. These species participated in the formation of the silicalite-1 shell and likely existed in extra-framework positions. TG analysis revealed that the hydrophobicity of Fe–ZSM-5 could be improved by the covered silicalite-1, the adsorption capability of H_2_O decreased with the increasing content of silicalite-1 shell. Compared to the Fe–ZSM-5, the resultant core–shell zeolites exhibited significantly enhanced NO_x_ conversion in the selective catalytic reduction of nitrogen oxides with ammonia (NH_3_-SCR), particularly for CS_1_ and CS_2_. Additionally, when 10% H_2_O was introduced into the reaction mixture, the NO_x_ conversion of Fe-ZSM-5 declined immediately, whereas the activities of the core–shell zeolites were hardly influenced, indicating their excellent resistance to water. The excellent catalytic activity of core–shell zeolites should be attributed to the adsorption of NO_x_ on silicalite-1 shell, Fe species transformation during hydrothermal crystallization, and the hydrophobicity of silicalite-1. The authors also suggested that controlling the thickness of the silicalite-1 shell is essential in the preparation of core–shell materials, as an excessively thick shell may increase diffusion resistance and impair catalytic performance, such as the CS_3_ sample.

**Figure 19:**
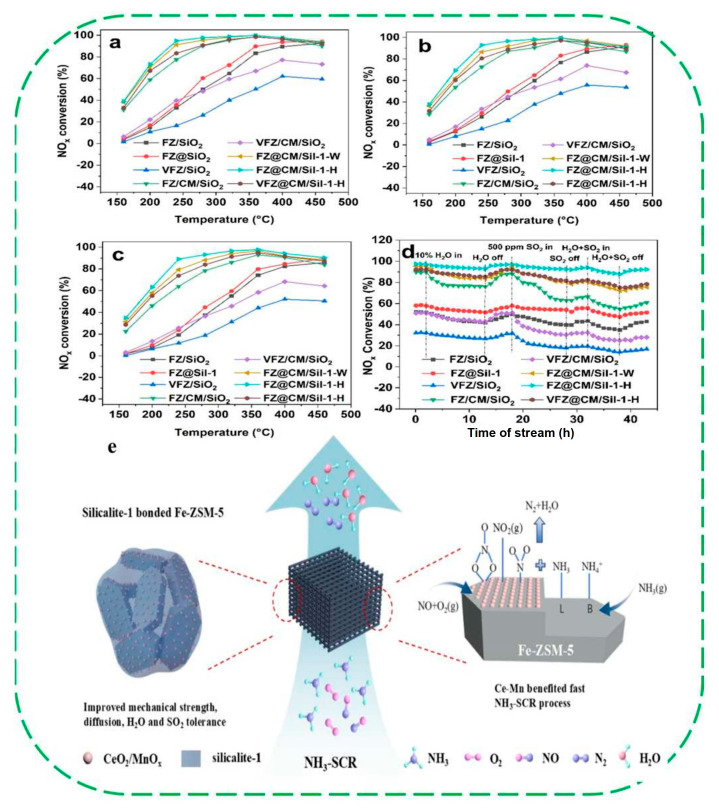
NO_x_ conversion of monoliths at GHSV = 30,000 (**a**), 50,000 (**b**) and 70,000 mL⋅g^−1^⋅h^−1^ (**c**), and stability test of monoliths at 300 °C and GHSV = 50,000 mL⋅g^−1^⋅h^−1^ (**d**). Reaction conditions: 500 ppm NO, 500 ppm NH_3_, 5% O_2_. (**e**) The proposed structure-function relationship of FZ@CM/Sil-1-H monolith for NH_3_-SCR. Reproduced with the permission of ref. [99]. Copyright 2025, Elsevier.

To further improve the catalytic performance of NH_3_-SCR, Tian et al. recently reported the fabrication of robust, binder-less Ce–Mn doped Fe–ZSM-5 monoliths (denoted as FZ@CM/Sil-1-H) via a sequential process combining three-dimensional (3D) printing with conversion of the SiO_2_ binder into the silicalite-1 phase through the H_2_O_2_ assisted dry-gel conversion (DGC) [99]. It should be noted here that the structure of FZ@CM/Sil-1-H is very similar to that of the core–shell catalyst mentioned above. Water contact angle measurements demonstrated an obvious increase in hydrophobicity after DGC, indicating that the transformation of SiO_2_ into silicalite-1 enhanced the hydrophobicity of monoliths. Compared to the sample prepared without H_2_O_2_ assistance (FZ@CM/Sil-1-W), FZ@CM/Sil-1–H showed superior resistance to higher space velocity and exhibited higher catalytic activity, with T_90%_ at gas hourly space velocity = 50,000 and 70,000 mL/g/h reduced 31 °C and 43 °C, respectively (Figure 19b,c). This result implied that the “adhesive plaster-like” silicalite-1 enhanced the diffusion of reactants and thus the NH_3_–SCR efficiency. After hydrothermal treatment under 10% H_2_O/air blowing at 750 °C for 16 h, the resulting VFZ@CM/Sil-1-H still showed similar catalytic activity to the parent FZ@CM/Sil-1-H, which can be attributed to its improved hydrophobicity. Furthermore, FZ@CM/Sil-1-H displayed the smallest loss in NO conversion during the H_2_O and SO_2_ tolerance tests, attributed to the uniform “adhesive plaster-like” silicalite-1 layer coated on the surfaces of Fe–ZSM-5 and the Ce–Mn species (Figure 19d), confirming the positive roles of H_2_O_2_-assisted DGC in enhancing the tolerance of the monoliths to H_2_O and SO_2_.

The authors further proposed a structure–function relationship for FZ@CM/Sil-1-H in NH_3_-SCR (Figure 19e). Firstly, converting the SiO_2_ binder into an “adhesive plaster-like” silicalite-1 phase simultaneously increased the mechanical strength of the monolith by binding zeolite particles together, increasing reactant diffusion through the increased S_BET_, and strengthening the tolerance to H_2_O and SO_2_ by shielding active sites, thus synthetically improving the NH_3_–SCR activity and stability of the monolith. Secondly, doping the Ce–Mn species enhanced the redox property and oxygen vacancy concentration of monoliths, thereby facilitating the conversion of NO into NO_2_, nitrite and nitrate. Thirdly, NH_3_ adsorbed on Brønsted or Lewis acid sites reacted with NO_2_, nitrite or nitrate, and enabling the establishment of the fast SCR reaction.

**Figure 20:**
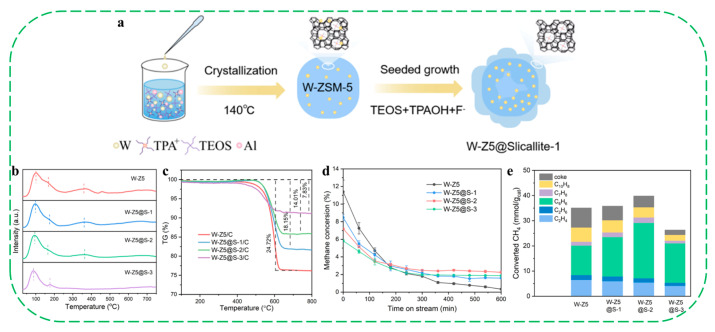
(**a**) Schematic illustration for the synthesis of W-Z5@Silicalite-1. (**b**) NH_3_–TPD curves of W–Z5, W–Z5@S-1, W–Z5@S-2, W–Z5@S-3 and (**c**) TG curves W–Z5/C, W–Z5@S-1/C, W–Z5@S-2/C, and W–Z5@S-3/C. (**d**) Methane conversion of W–Z5, W-Z5@S-1, W-Z5@S-2, and W-Z5@S-3 as a function of time on stream. (**e**) Relative distributions of hydrocarbon products and coke of W–Z5, W–Z5@S-1, W–Z5@S-2, and W–Z5@S-3. Reproduced with the permission of ref. [100]. Copyright 2025, Elsevier.

Similarly, Jia et al. synthesized a series of W-modified ZSM-5 nanoparticles encapsulated by silicalite-1 shells of varying thicknesses (W-Z5@S-X, Figure 20a) [100]. The optimized W-Z5@S-2 catalyst achieved an optimal balance between methane conversion (7.2%) and aromatic selectivity (56.3%), yielding 22 mmol gcat^−1^ of benzene over 600 min in methane dehydroaromatization reaction (Figure 20d,e). The excellent catalytic performances of W-Z5@S-2 catalyst could be mainly attributed to the thermal robustness of the W active species and controlled surface Brønsted acidity concentration by the silicalite-1 shell. Combining with the NH_3_–TPD and TG analysis, the authors found that the epitaxial growth of the silicalite-1 layer effectively passivated Brønsted acid sites on the surface of W-ZSM-5 core, which can effectively reduce coke formation (68%) without compromising methane diffusion (Figure 20b,c,e).

In another work reported by Xing and coworkers, they found that a silicalite-1 shell-coated Co/ZSM-5 microcapsule catalyst (Co/ZSM-5@Silicalite-1) exhibited very high efficiency for the direct conversion of syngas to gasoline range hydrocarbons, with 68.9% CO conversion and 74.7% hydrocarbon selectivity; this was much better than the Co/ZSM-5 catalyst prepared by the incipient-wetness impregnation method or physically mixed approach [101]. The authors believed that the hydrophobic channels of silicalite-1 shell should be responsible for the relatively high olefin content, since it favors the reverse water–gas shift reaction, thus significantly inhibiting the formation unfavorable side product of CO_2_. Furthermore, the silicalite-1 shell is suitable for diffusion of reactants to access the core, and may also considerably contribute toward stability against coke deposition on metal sites. In a later work, the same group also employed this approach to obtain a series of silicalite-1 coated Fe/ZSM-5 microcapsule catalysts (Fe/ZSM-5@S1) [102]. The water droplet test confirmed the transformation of the hydrophilic surface of the ZSM-5 supported core to a hydrophobic surface through silicalite-1 coating. The condition-optimized Fe/ZSM-5@S1 catalyst (with double silicalite-1 layer) showed enhanced selectivity for direct synthesis of hydrocarbons from syngas, which can also be assigned to the hydrophobic nature of the composite catalyst. As proposed by authors, these microcapsule catalysts might be considered to be utilized on a commercial scale, due mainly to its facile preparation method and promising catalytic performance for producing clean fuels.

The above results shows that core–shell architectures represent a unique class of multifunctional materials capable of tandem catalysis, in which synergistic effects between the core and shell can lead to remarkable enhancements in catalytic performance. In some cases, the core–shell structured catalyst can be prepared through relatively simple methods (e.g., via hydrothermal synthesis), thus showing great potential for commercial production and industrial applications. However, in most cases, the synthesis of such composite catalysts commonly involves multiple steps, including core preparation, shell coating, and secondary crystallization, which can result in the increase in production time, energy consumption and overall cost. Nevertheless, the development of hydrophobic core–shell zeolite catalysts is still highly desirable, since it may provide new reaction pathways for the efficient synthesis of high-value-added chemicals. Future research should focus on developing greener and more efficient preparation processes to enhance the sustainability and practical value of this type of catalyst system.

## 5. Conclusions

In this review, we summarized the recent progress on the improvement of the hydrophobicity of zeolite-based catalysts for promoted application in various industrially important catalytic processes. By using suitable regulation strategies, appropriate hydrophobic environments might be constructed for meeting the requirement of a given catalytic reaction. The main research advancements highlight that optimizing the hydrophobicity environment of zeolite-based catalysts may have a positive impact on the following aspects: (1) improving reactant enrichment near active sites; (2) suppressing competitive water adsorption; (3) facilitating product desorption for shifting the reaction balance; and (4) enhancing the structure stability of zeolites in aqueous or humid conditions. These results clearly demonstrate the great potential of the hydrophobic zeolite-based catalysts for the application in the field of industrial catalysis.

In order to promote the application and development of hydrophobic zeolite-based catalysts, more efforts are still required in future work. Firstly, it is necessary to develop more simple and effective regulation strategies to optimize the hydrophobic environment of the zeolite-based catalysts. Specifically, the usage of some expensive or toxic agents like fluorides should be avoided as far as possible.

Secondly, the stability of those hydrophobic zeolite catalysts, which are derived from the post-modification with organic functional groups (e.g., silanization modification), needs to be strengthened since they may decompose easily during the catalytic reaction and regeneration process (e.g., to remove coke by calcination). In this case, it would be very significant if a simple and mild regeneration method could be developed for removing coke or recovering the catalytic performance of the hydrophobic zeolites. Another interesting subject might be the combination of zeolitic framework composition adjustment and post-modification strategies. This integrated approach may effectively optimize the hydrophobic environment while preserving sufficient active sites, potentially leading to remarkable synergistic effects, thereby opening new possibilities for the design of zeolite-based catalyst materials with tailored hydrophobic functionality.

Thirdly, it would be very attractive to integrate the hydrophobic zeolites with other functional nanoparticles or single-atom active sites for efficiently achieving multi-step catalytic reactions, or to combine the hydrophobic character with the structure/morphology optimization of zeolite crystals for generating synergistic regulation effects on enhancing the catalytic performance in a given reaction. Beyond traditional heterogeneous catalysis, the integration of hydrophobic zeolites with emerging fields should present groundbreaking opportunities. For instance, in electrocatalysis, the ability of hydrophobic zeolites to modulate interfacial water environments could enhance the efficiency of key reactions such as oxygen reduction or hydrogen evolution. In plastic upcycling, hydrophobic channels in zeolites may facilitate the diffusion of bulky polymer-derived intermediates and promote selective C–C bond cleavage. Looking ahead, underexplored directions such as blue hydrogen purification and energy storage may further underscore the broader potential of hydrophobicity engineering in zeolites and inspire cross-disciplinary innovations.

Fourthly, more characterization means and theory calculation should be conducted to accurately describe the hydrophobic microenvironment. For instance, a couple of operando techniques such as ultrafast cameras, magnetic resonance imaging (MRI), in situ liquid atomic force microscopy (AFM) and neutron scattering could be conducted for visualizing the diffusion behavior of a reactant or product in the given zones of catalysts under the test reaction conditions. This would be helpful for directly measuring the states and mass transport dynamics under working conditions, and would be more powerful when theoretical calculations and simulations are combined to deeply understand the effect of hydrophobicity on the catalytic performance of the zeolite-based catalysts.

In short, there is still a very large opportunity to precisely tune the hydrophobic environment of zeolite-based catalysts to meet the urgent need in chemical industries and environment remediation. It can be expected that more efficient hydrophobic zeolite-based catalysts will be developed by adopting new and effective preparation/regulation strategies, which will certainly promote the basic studies and practical application of the functionalized zeolite-based catalysts with optimized hydrophobic environment.

## Figures and Tables

**Table 1:** Comparison of standardized indexes (HF, carbon deposition and surface hydroxyl groups) and catalytic performance (deactivate time and PO selectivity) of different samples [55].

Catalysts	HF ^a^	Carbon Deposition (%) ^b^	Stability (h)	Surface Hydroxyl Groups (Q^3^/Q^4^,%) ^c^	PO Selectivity (%) ^d^
Au/MTS-1-8	0.056	5.65	6	14.68	~60
Au/MTS-1-10	0.067	3.52	13	11.18	~60
Au/MTS-1-12	0.074	3.10	17	9.15	~78
Au/MTS-1-16	0.084	1.94	25	8.92	~90

^a^ calculated according to the following equation: HF = (V_micro_/V_total_) × (S_meso_/S_BET_). ^b^ Calculated by TG profiles after reaction 25 h. ^c^ Calculated by ^29^Si MAS NMR spectra. ^d^ after reaction 25 h.

**Table 2:** Catalytic activity of various catalysts for Baeyer–Villiger oxidation of 2-adamantanone with hydrogen peroxide, and stability data (regeneration way, cycles, and activity retention) [58] ^a^.

Catalyst	Si/Sn ^b^	Cover. (%) ^c^	Lactone sel. (%)	TOF (h^–1^) ^d^	STY (h^–1^) ^e^	Regeneration Methods	Cycles	Cover. (%) ^f^	Activity Retention (%) ^g^
blank	-	0	-	0	0	-	-	-	-
Beta–Re	∞	0	-	0	0	-	-	-	-
SnO2/Beta–Re	30	0.3	91.1	0.4	0.04	-	-	-	-
Sn–Beta–Re-150	152	26.9	99.5	195	3.6	-	-	-	-
Sn–Beta–Re-60	62	49.7	99.3	147	6.7	-	-	-	-
Sn–Beta–Re-30	33	61.6	99.6	98	8.3	calcination	4	~59	96
Sn–Beta–F-150	153	16.6	99.1	123	2.2	calcination	4	~15	90
Sn–Beta–GPS-36	36	48.6	99.5	84	6.4	calcination	4	~38	78
Sn–Beta–SSIE-30	30	47.3	99.3	73	6.2	calcination	4	~34	72

^a^ Reaction conditions: cat, 50 mg; 2-adamantanone, 2 mmol; H_2_O_2_ (30 wt %), 4 mmol; chlorobenzene, 10 mL; temp., 363 K; time, 0.5 h. ^b^ Molar ratio determined by ICP. ^c^ Conv. = moles of 2-adamantanone converted/initial moles of 2-adamantanone × 100%. ^d^ Turnover frequency (TOF), moles of lactone produced hourly per mole of Sn active sites in catalysts. ^e^ Space-time yield (STY), grams of lactone produced hourly per gram of catalysts. ^f^ Conv. (after 5 cycles) = moles of 2-adamantanone converted/initial moles of 2-adamantanone×100%. ^g^ Activity retention (%) = (f)conversion of ketone after 5 cycles/(c)initial conversion of ketone.

## Data Availability

No new data were created or analyzed in this study.
